# Modeling Binary and Multicomponent Systems Containing Supercritical CO_2_ with Polyethylene Glycols and Compounds Relevant to the Biodiesel Production

**DOI:** 10.3390/molecules27185785

**Published:** 2022-09-07

**Authors:** Ioannis Tsivintzelis, Georgios Koutsou, Georgios M. Kontogeorgis

**Affiliations:** 1 Department of Chemical Engineering, Aristotle University of Thessaloniki, GR-54124 Thessaloniki, Greece; 2 Center for Energy Resources Engineering (CERE), Department of Chemical and Biochemical Engineering, Technical University of Denmark, DK-2800 Kgs. Lyngby, Denmark

**Keywords:** supercritical CO_2_, biodiesel, triglycerides, polyethylene glycols, glycerol, CPA, phase equilibrium

## Abstract

The CPA equation of state is applied to model binary, ternary, and multicomponent mixtures that contain CO_2_ with polyethylene glycols or compounds relevant to biodiesel production, such as glycerol and various triglycerides. Effort has been made to evaluate the model performance on correlating both the liquid and the vapor phase compositions, which is a demanding task, revealing the model’s and parameters’ limitations, due to the rather low concentrations of heavy compounds in the vapor phase. Initially the model’s binary parameters, which in all cases were temperature independent, were estimated using experimental data for binary systems. Those parameters were used to predict the phase behavior of supercritical CO_2_ containing ternary and multicomponent mixtures. Since no parameter was adjusted to ternary or multicomponent systems’ data, the reported CPA results for such mixtures are considered as pure predictions. This is the final part of a series of studies [Tsivintzelis et al. Fluid Phase Equilibria 430 (2016) 75–92 and 504 (2020) 112337] that complete the parameterization of the CPA equation of state for systems relevant to the biodiesel production, which allows the application of the model to multicomponent mixtures of the relevant processes.

## 1. Introduction

The processing of vegetable seeds and oils with supercritical CO_2_ has attracted significant attention mainly because the relevant extraction or purification processes require lower temperatures, while CO_2_ is nontoxic, and it is easily removed from the products simply by depressurizing the system. Such pressure-induced separation diminishes the need for adequate thermal separations, as in the traditional processes with liquid and often toxic solvents. Another important advantage is that the solubility of other substances in such a supercritical solvent is strongly affected by moderate changes in temperature and pressure, rendering the solvent power of supercritical CO_2_ as a tunable parameter that can be adjusted to the needs of specific processes. This allows its successful use in various demanding separations of high-value compounds from mixtures of similar fluids, such as the separation of antioxidants from natural products. Despite the disadvantage of higher capital and operational costs imposed by the use of high pressures, the aforementioned advantages are often crucial in the food, cosmetic, pharmaceutical, and nutraceutical industries [1,2,3].

However, vegetable oils are also used to produce biodiesel, whose production volume increases following the need for replacing traditional fossil fuels with fuels from renewable sources. Biodiesel is produced through the transesterification reaction of various vegetable oils with methanol (or ethanol), which results in multicomponent mixtures containing methyl (or ethyl) esters of fatty acids and glycerol as a byproduct. Recently, the supercritical CO_2_ was suggested as a reaction medium or as a co-solvent for the transesterification reaction and the purification of biodiesel, i.e., removal of glycerol and other contaminants [4].

Despite considering supercritical CO_2_ as a valuable solvent or reaction medium, and despite its nontoxic character, such fluid, when massively released to the atmosphere from the combustion of fossil fuels, is considered as an environmental threat due to its action in the greenhouse phenomenon. Thus, an important research effort has been made to develop processes for capturing CO_2_ from flue gases, which should be economically feasible and environmentally friendly. In this direction, polyethylene glycols and various alcohols have been suggested, among a vast amount of other potential solvents, for the physical absorption of CO_2_ [5].

Despite the differences of the three groups of CO_2_ processes mentioned above, i.e., extraction and purification of natural products, biodiesel production, and CO_2_ capture with physical absorption, all of them require the successful prediction of the phase behavior of binary and multicomponent CO_2_ mixtures that, among others, may contain glycols, glycerol, low-molecular-weight alcohols, water, glycerides, fatty acids, and alkyl esters of fatty acids.

Modeling the thermodynamic properties of such mixtures is rather demanding, mainly due to the non-ideal behavior of some systems (i.e., mixtures with organic acids and other hydrogen-bonding or high-molecular-weight compounds) and the use of high pressures [6,7,8]. In such systems, the hydrogen-bonding behavior significantly affects the phase behavior and other thermodynamic properties, especially the vaporization enthalpies and the heats of mixing [6,7,8]. Thus, explicitly accounting for various self- and cross-association interactions is the safest way for modeling using equations of state. However, as mentioned in the first two studies of this series of articles, in many cases, there is a lack of accurate experimental data, which renders difficult the parameterization and the evaluation of thermodynamic models [7,8]. In more detail, measuring the critical properties, or the vapor pressures, at relatively high temperatures of such high-molecular-weight compounds (i.e., fatty acids, glycerides and esters of fatty acids) is often non feasible due to decomposition. Furthermore, most often, those compounds are produced in mixtures with various isomers or similar substances, and thus, the experimental data refer to samples of low purity. Finally, many mixtures of interest, such as systems with alcohols and fatty acids, glycerides, or esters, are reactive, rendering the experimental investigation of their phase behavior rather demanding. For this reason, many contradictory data are often found in literature despite the fact that many experimental data of good quality were measured during the last years.

The CPA equation of state [9] was used to describe a vast number of fluids as recently reviewed [10,11]. In addition, glycol-containing systems, including the demanding glycol–water LLE, were modeled [12,13,14,15,16]; however, the polyethylene glycols have not been studied so far. Biodiesel systems, mainly at low pressures, have been modeled by Coutinho and co-workers in various studies [17,18,19,20,21,22,23,24,25], which in many cases contain correlations of pure and binary CPA parameters allowing predictions for systems for which there is lack of experimental data, while many of such studies also contain experimental data. Finally, systems at high pressures containing CO_2_ with water, alcohols, glycols, and/or hydrocarbons were extensively studied [15,26,27,28,29,30,31].

This study is the final part of a series of articles [7,8]. In the first part [7], the CPA pure fluid parameters for various compounds such as glycerol, heavy esters, fatty acids, and glycerides were presented, along with correlations that allow the prediction of pure fluid parameters in cases of lack of experimental data (such correlations were used in this study in case of triolein, as described in Section 3.1). Moreover, binary and multicomponent mixtures containing alcohols, water, glycerol, fatty acids, heavy esters, or glycerides were considered. In the second part [8], the model was applied to describe binary mixtures of CO_2_ with fatty acids and their methyl- or ethyl-esters, while a limited number of multicomponent systems was considered. However, no glycerol- or glyceride-containing systems were studied. In both previous studies, correlations of the binary parameters are presented, allowing the prediction of their values in cases of lack of experimental data. Some multicomponent biodiesel systems were recently modeled [30].

In this study, the CPA equation of state was applied for first time to polyethylene glycols of rather low polymer molecular weight, and correlations of the pure fluid parameters are presented allowing the prediction of their values for various molecular weights. Then, the model was applied to binary systems that contain CO_2_ with polyethylene glycols, glycerol, or various glycerides, and the binary parameters were obtained. Using binary parameters optimized solely to experimental data for binary mixtures, the predicting ability of the model was evaluated for ternary and multicomponent systems.

## 2. The CPA Equation of State

The cubic-plus-association (CPA) equation of state (EoS) is a combination of the SRK EoS and the association term of the SAFT type models [9,10,11]. Thus, this model is capable of describing systems with hydrogen-bonding fluids such as water, alcohols, and glycols. The EoS, in terms of pressure, is given by the following equation:

(1) $$P=\frac{RT}{V_{m}-b}-\frac{\alpha \left(T\right)}{V_{m}\left(V_{m}+b\right)}-\frac{1}{2}\frac{RT}{V_{m}}\left(1+\rho \frac{\partial lng}{\partial \rho }\right)\sum_{i}x_{i}\sum_{A_{i}}\left(1-X_{A_{i}}\right)$$

where *x_i_* is the mole fraction of component *i*, and *X_Ai_* is the fraction of the free (not associated with other sites) sites of type *A* on molecule *i*, related to the association strength Δ*^AiBj^* as follows:

(2) $$X_{A_{i}}=\frac{1}{1+\rho \cdot \sum_{j}x_{j}\sum_{B_{j}}\left(X_{B_{j}}\cdot \Delta^{A_{i}B_{j}}\right)}$$

(3) $$\Delta^{A_{i}B_{j}}=g\left(\rho \right)\left[exp\left(\frac{\varepsilon^{A_{i}B_{j}}}{R\cdot T}\right)-1\right]b_{ij}\cdot \beta^{A_{i}B_{j}}$$

where g(ρ)=1/(1−1.9n) is the radial distribution function, n=(1/4)bρ the reduced density, *ρ* the molar density, *ε^AiBj^* the association energy, *β^AiBj^* the association volume, and $b_{ij}=\left(b_{i}+b_{j}\right)/2$. The co-volume parameter, *b_i_*, of component *i* is considered temperature independent.

A Soave-type relationship is used for the interaction energy of the physical term, as follows:

(4) $$a_{i}\left(T\right)=a_{0,i}{\left[1+c_{1,i}\cdot \left(1-\sqrt{T_{r,i}}\right)\right]}^{2}$$

where *a*_0_ and *c*_1_ are pure fluid parameters, and *T_r_* is the reduced temperature.

The next mixing and combining rules are used for the SRK physical term [9], whereas the one temperature-independent binary interaction parameter, *k_ij_*, was used according to the following relationships:

(5) $$\alpha =\sum_{i}\sum_{j}x_{i}x_{j}\alpha_{ij}$$

(6) $$\alpha_{ij}=\sqrt{\alpha_{i}\alpha_{j}}\left(1-k_{ij}\right)$$

(7) $$b=\sum_{i}x_{i}\cdot b_{i}$$

For interactions between two self-associating fluids, *i* and *j*, the CR-1 rule is used through the following relationships:

(8) $$\varepsilon^{A_{i}B_{j}}=\frac{\varepsilon^{A_{i}B_{i}}+\varepsilon^{A_{j}B_{j}}}{2},\;\beta^{A_{i}B_{j}}=\sqrt{\beta^{A_{i}B_{i}}\cdot \beta^{A_{j}B_{j}}}\;$$

For interactions between one self-associating with one non self-associating fluid, the modified CR-1 (mCR-1) combining rule is used [31]:

(9) $$\varepsilon^{A_{i}B_{j}}=\frac{\varepsilon^{A_{i}B_{i}}+\varepsilon^{A_{j}B_{j}}}{2},\;\beta^{A_{i}B_{j}}=adjustable$$

## 3. Results and Discussion

### 3.1. CPA Pure Fluid Parameters

For non-associating pure fluids, the CPA EoS is reduced to the SRK model and requires the knowledge of three pure fluid parameters, which, in contrast with the SRK EoS, are not estimated form the critical properties but are adjusted to pure fluid experimental vapor pressures and liquid densities. In cases of self-associating fluids, the application of the model requires the knowledge of two additional parameters, namely the association energy and the association volume, which may be simultaneously adjusted with the three physical parameters, but they can also be adopted form experimental values or ab initio calculations [31].

**Figure 1:**
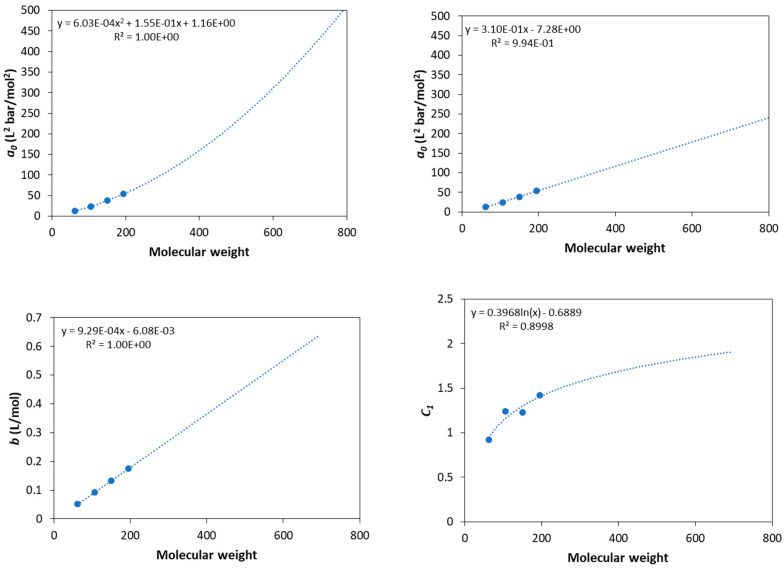
Correlations of pure fluid parameters for glycols and extension to higher molecular weights.

All the pure fluid parameters needed for calculations of this study were adopted from the literature except for the parameters of glycols, which were estimated by adjusting the model predictions to DIPPR data for vapor pressures and liquid molar volumes [32]. All parameters used in this study are presented in Table 1. For glycerol, the 3 × 2B association scheme is used to describe the self-associating behavior since glycerol contains three hydroxyl groups in each molecule [7]. In addition, here, it is worth mentioning that the parameters of triolein were predicted using the correlations presented by Tsivintzelis et al. [7] due to lack of (confirmed) experimental data.Since experimental data for vapor pressures do not exist for various polyethylene glycols, the estimation of the CPA pure fluid parameters becomes difficult. Many studies showed that the estimation of pure fluid parameters of various models, including the SAFT type ones and lattice fluid theories, by using only volumetric properties does not result in optimum parameters for phase equilibrium calculations of binary systems [33]. For this reason, various approaches to predict such parameters were developed, including group contribution methods [33,34,35]. Using the CPA model for compounds of the same family, it was shown that the co-volume parameter, *b*, is a linear function of molecular weight (or the van der Waals volume of the fluid), the *c*_1_ parameter reaches a plateau for relatively high molecular weights, while the energetic parameter, *a*_0_, of the physical term shows a linear or a mild polynomial dependence from molecular weight (or the van der Waals volume of the fluid) [7,17,28]. However, it was shown that appropriate correlations of the three physical CPA parameters are facilitated when the association parameters are kept constant for all fluids of the same family since the two energetic parameters, i.e., the *a*_0_ and *ε* parameters, are highly correlated to each other [7]. Having in mind such findings for the CPA model, correlations of the pure fluid parameters for glycols were estimated as presented in Figure 1. For *a*_0_, both a linear and a second-order polynomial dependence was used. Such correlations allow for the prediction of pure fluid parameters for polyethylene glycols of various molecular weights, as shown in Table 2. The critical temperature reported in Table 2 was predicted using the Joback’s group contribution method for the closest ethylene glycol [36].

### 3.2. CO_2_–Polyethylene Glycols VLE

Next, the model was applied to describe the solubility of CO_2_ in polyethylene glycols of various molecular weights. In all cases, the *a*_0_ was adopted from the linear correlation (see Figure 1 and Table 2) since it was shown that it gives slightly more accurate results, especially for higher molecular weights.

Tsivintzelis et al. modeled the CO_2_–glycol vapor–liquid equilibrium (in systems containing ethylene glycol, diethylene glycol, and triethylene glycol) using two major approaches, i.e., assuming CO_2_ as inert compound and assuming that CO_2_ has one site that is able to cross-associate with glycols [15]. Using the first approach, the model satisfactorily describes the liquid phase compositions; while only using the second approach (and, consequently, using one extra binary parameter) the model satisfactorily describes the vapor phase compositions. However, since in systems with polyethylene glycols, the liquid phase is of interest; the first approach was used in this study for simplicity, and CO_2_ was modeled as inert compound.

**Figure 2:**
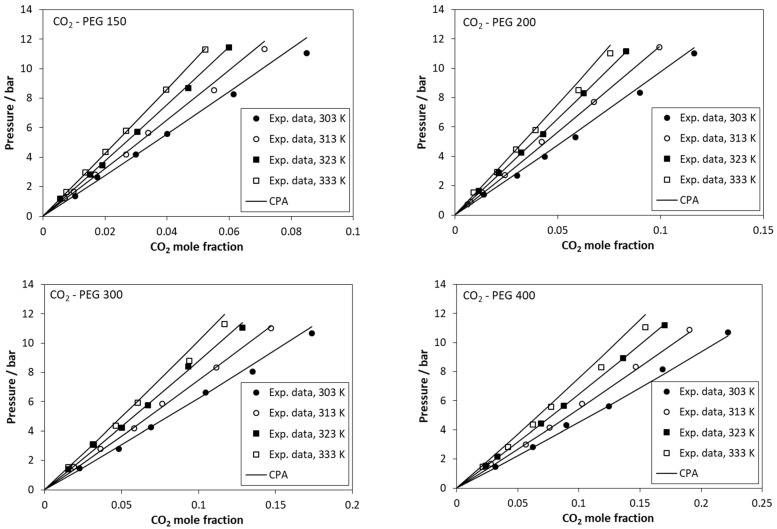
Solubility of CO_2_ in polyethylene glycols. Experimental data (points [38]) and CPA calculations (lines) using the *k_ij_* values of Table 3.

The results using one temperature-independent binary interaction parameter, *k_ij_*, are presented in Table 3 and in Figure 2. In all cases, the CPA correlations show a satisfactory agreement with the experimental data. The values of the binary interaction parameters are low and positive for low molecular weights, while they become negative for higher molecular weights. Since increasing the molecular weight, the strong intermolecular interactions are expected to decrease, the negative values of *k_ij_* for higher molecular weights mainly reflect the inaccuracies of PEG’s pure fluid parameters due to the approximate way of their estimation, i.e., prediction using the correlations of Figure 1.

### 3.3. CO_2_–Glycerol Binary System

In contrast to polyethylene glycols, glycerol is a low-molecular-weight compound. It has three hydroxyls in each molecule, and consequently, it presents considerable hydrogen bonding. Furthermore, Tsivintzelis et al. showed that systems containing CO_2_ and alcohols are better described by the CPA model if strong specific interactions between the positively charged carbon atom of CO_2_ and the oxygen of the hydroxyl group are taken into consideration, as indicated by various ab initio studies, assuming one site that is able to cross-associate with alcohols on every CO_2_ molecule [29,31]. In addition, only using the same approach for CO_2_–low-molecular glycol systems, the very small concentration of glycols in the vapor phase can be adequately described [15].

**Figure 3:**
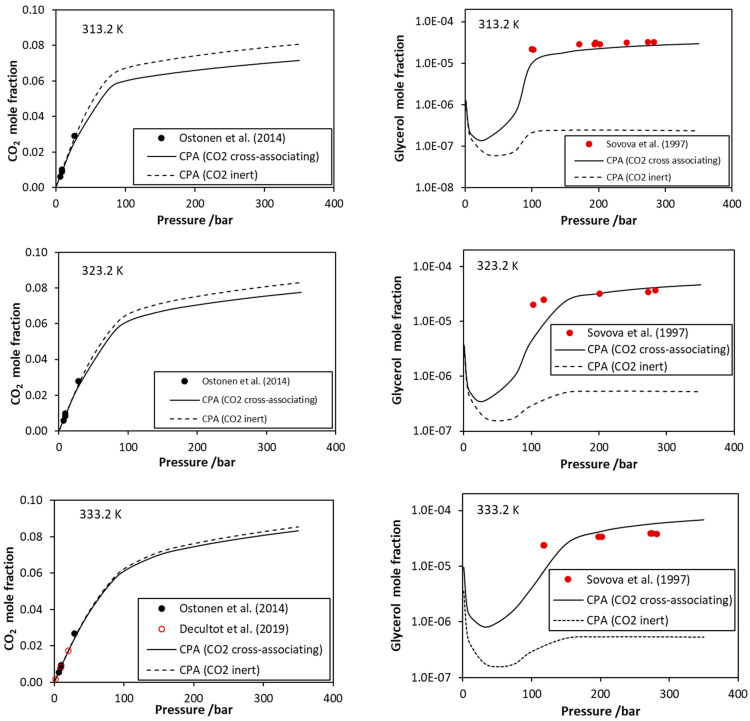
CO_2_–glycerol VLE. CO_2_ mole fraction in the liquid phase and glycerol mole fraction in the vapor phase. Experimental data [39,40,41,42] and CPA correlations using the interaction parameters of Table 4.

In this work, the CO_2_–glycerol VLE was modeled using two approaches, i.e., assuming CO_2_ as inert compound and, secondly, assuming one positive site on every CO_2_ molecule that is only able to cross-associate with glycerol’s negative sites (the 3 × 2B association scheme was used for glycerol). Thus, only the binary interaction parameter (*k_ij_*) was adjusted in the first case, while both the *k_ij_* and the cross-association volume (*β_cross_*) were optimized in the second one (using the mCR-1 rule). The obtained binary parameters and the deviations of model calculations from experimental data are presented in Table 4. Some representative results are presented in Figure 3, while more results are shown in Figures S1–S6 of the supplementary information file. Similar conclusions to CO_2_–glycol mixtures [15] were obtained; i.e., using a single binary interaction parameter, the model satisfactorily describes the liquid phase, but only considering CO_2_ as a cross-associating compound and, consequently, using two adjustable binary parameters, the model satisfactorily describes the vapor phase compositions. Thus, such a mixture that presents various self- and cross-hydrogen-bonding interactions is better described if, in the CPA model, all possible association interactions are explicitly accounted for.

Here, it is worth mentioning that, as shown by the CPA correlations, the glycerol mole fraction in the vapor phase presents a minimum when plotted against pressure (Figure 3). This resembles the minimum that is observed for the CO_2_–water system and reflects the existence of vapor–liquid–liquid equilibrium in relatively low temperatures. Similarly to the minimum of CO_2_–water system, such behavior of the CO_2_–glycerol mixture, predicted by the model, may be of interest in various separation processes.

### 3.4. CO_2_–Glycerides

The phase behavior of five CO_2_–triglyceride mixtures containing tricaprylin, tributyrin, trilaurin, trimyristin, and triolein was investigated. In all cases, both CO_2_ and triglycerides were modeled as inert compounds, and only one temperature-independent binary parameter, *k_ij_*, was used. The obtained binary parameter and the deviations of model calculations from experimental data are presented in Table 5. Overall, it was observed that the modeling of such mixtures present significant difficulty, mainly due to the very low concentration of the heavy compound (triglyceride) in the vapor phase. Forcing the model to satisfactorily describe the vapor phase increases the deviations for the liquid phase compositions. Consequently, in all cases the model correlations for the CO_2_ mole fraction of the liquid phase range between 5–11%.

**Figure 4:**
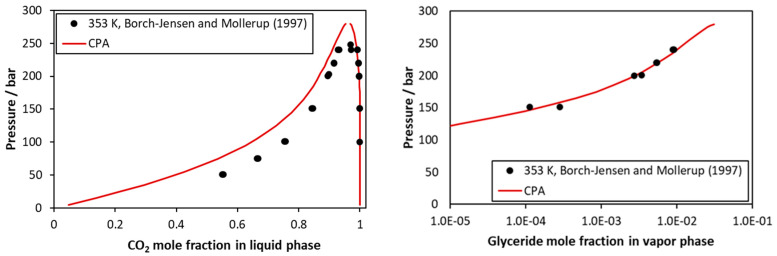
CO_2_–tricaprilyn VLE at 353 K. Experimental data [43] and CPA correlations using the binary interaction parameter of Table 5.

In more detail, considering the CO_2_–tricaprylin system, representative calculations are presented in Figure 4 and in Figures S7 and S8 of the supplementary information file. The model represents very satisfactorily the very low vapor phase content of tricaprylin but underestimates to a low extent the CO_2_ mole fraction of the liquid phase.

**Figure 5:**
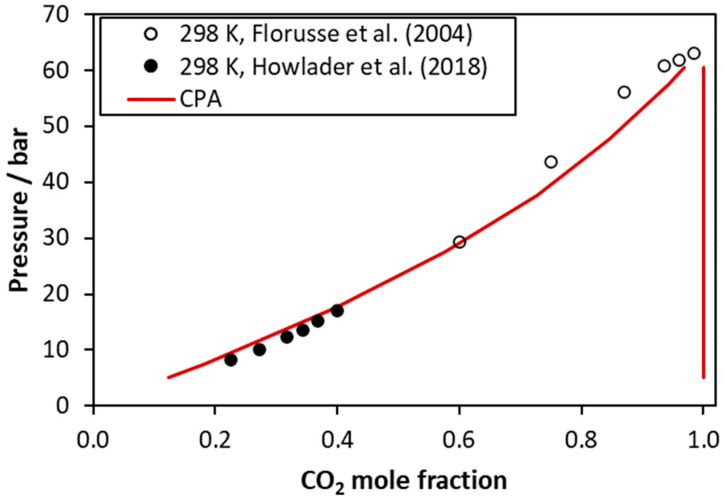
CO_2–_tributyrin VLE at 298 K. Experimental data [44,45] and CPA correlations using the binary interaction parameter of Table 5.

Results for the CO_2_–tributyrin are shown in Figure 5 and Figures S9–S11 of the supplementary information file. For this system, the binary interaction parameter was adjusted solely to liquid phase data since data for the vapor phase are not available. The model shows satisfactory agreement with the experimental data.

**Figure 6:**
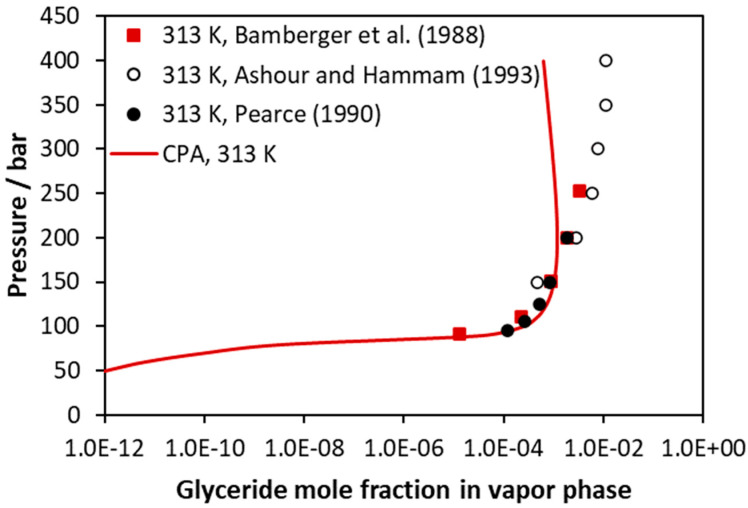
CO_2_–trilaurin VLE at 313 K (vapor phase compositions). Experimental data [46,48,49] and CPA correlations using the binary interaction parameter of Table 5.

Results for the CO_2_–trilaurin system are illustrated in Figure 6 and in Figures S12–S14 of the supporting information file. For this system, only one set of data for liquid phase compositions is available (see Figure S14 of the supporting information file), and consequently, the binary parameter was regressed using mainly vapor phase compositions, which, however, are available for high pressures. It is shown that the model satisfactorily describes the solubility of the glyceride in the vapor or the supercritical fluid phase up to approximately 200 bar, while higher deviations are observed for higher pressures.

**Figure 7:**
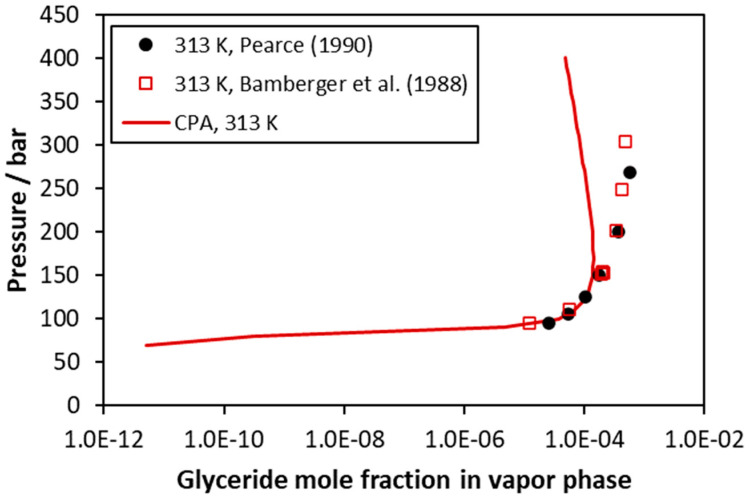
CO_2_–trimyristin VLE at 313 K (vapor phase compositions). Experimental data [46,48] and CPA correlations using the binary interaction parameter of Table 5.

Representative calculations for the CO_2_–trimyristin system are presented in Figure 7 and in Figures S15 and S16 of the supporting information file. No liquid phase compositions are available for this system, and consequently, the binary interaction parameter was adjusted only to vapor phase compositions. Similarly, for the CO_2_–trilaurin system, the model satisfactorily describes the very small solubility of trymyristin in the fluid phase up to approximately 200 bar, while higher deviations are observed at higher pressures.

**Figure 8:**
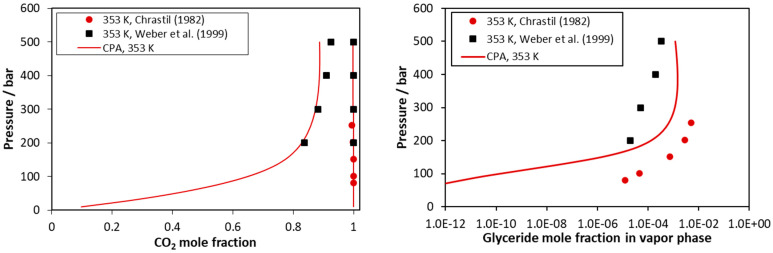
CO_2_–triolein VLE at 353 K. Experimental data [51,53] and CPA correlations using the binary interaction parameter of Table 5.

Results for the CO_2_–triolein mixture are presented in Figure 8 and in Figures S17–S20 of the supplementary information file. As presented in these Figures, the experimental data are scattered, while for the glyceride content of the vapor phase, data from different sources may differ up to two orders of magnitude. As presented in Table 5, the model correlations present an average deviation form experimental glyceride mole fraction of the vapor phase equal to 139%, but to a large extent, such value is attributed to the scattering of the experimental data, and Figure 8 and Figures S17–S20 present the capabilities of the model in a more realistic way. Furthermore, the reader should have in mind that, as mentioned above, the triolein pure fluid parameters were predicted using appropriate correlations in absence of (confirmed) experimental data.

### 3.5. Ternary and Multicomponent Systems

Next, the model was applied to predict the phase behavior of ternary and multicomponent mixtures and the results were compared to experimental data from literature [55,56,57,58,59,60]. In all cases, no parameter was regressed using ternary or multicomponent systems data, but all binary parameters were adopted from the respective sub-binary mixtures. Consequently, the calculations that are presented in this section are considered as pure predictions. All the used binary parameters are presented in Table 6.

Initially, the model was applied to describe the vapor-liquid equilibrium of mixtures containing CO_2_ with glycerol and methanol or ethanol. In all cases, one positive association site was assumed on every CO_2_ molecule that is only able to cross-associate with the negative sites of glycerol, methanol, or ethanol, and consequently, the modified CR-1 (mCR-1) combining rule was used for estimating the cross-association parameters [31]. Further, as shown in Table 6, the glycerol–methanol (or ethanol) cross-association parameters were calculated using the CR-1 combining rule [31].

**Figure 9:**
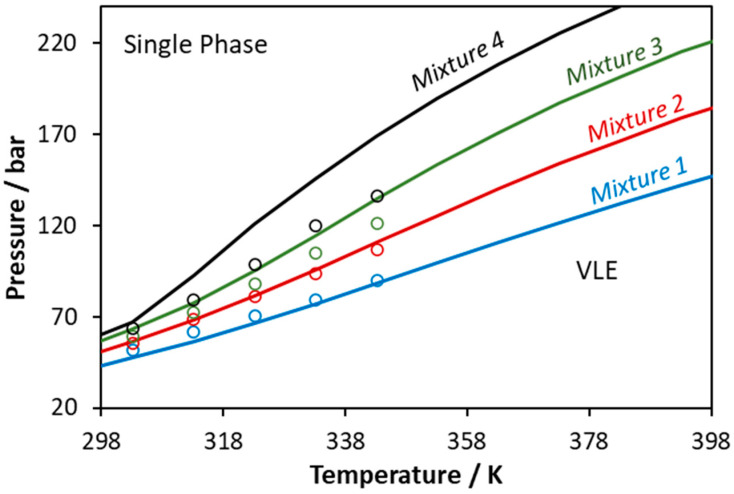
Phase boundaries for CO_2_–glycerol–methanol ternary system. Experimental data (points, [55]) and CPA predictions using the binary interaction parameters of Table 6 (Mixture 1: x_CO2_ = 0.3373, x_glycerol_ = 0.0212, x_methanol_ = 0.6415; Mixture 2: x_CO2_ = 0.4352, x_glycerol_ = 0.0180, x_methanol_ = 0.5468; Mixture 3: x_CO2_ = 0.5359, x_glycerol_ = 0.0148, x_methanol_ = 0.4493; Mixture 4: x_CO2_ = 0.6430, x_glycerol_ = 0.0114, x_methanol_ = 0.3456).

**Figure 10:**
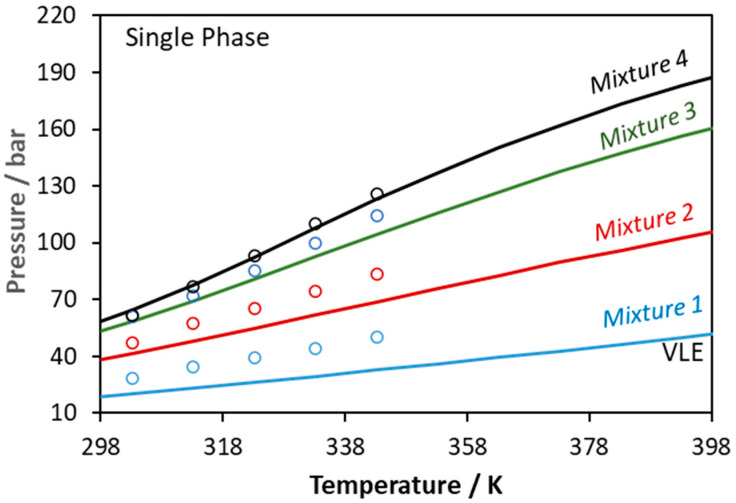
Phase boundaries for CO_2_–glycerol–ethanol ternary system. Experimental data (points, [56]) and CPA predictions using the binary interaction parameters of Table 6 (Mixture 1: x_CO2_ = 0.1333, x_glycerol_ = 0.0413, x_ethanol_ = 0.8254; Mixture 2: x_CO2_ = 0.2808, x_glycerol_ = 0.0342, x_ethanol_ = 0.6850; Mixture 3: x_CO2_ = 0.4276, x_glycerol_ = 0.0273, x_ethanol_ = 0.5451; Mixture 4: x_CO2_ = 0.4985, x_glycerol_ = 0.0239, x_ethanol_ = 0.4776).

**Figure 11:**
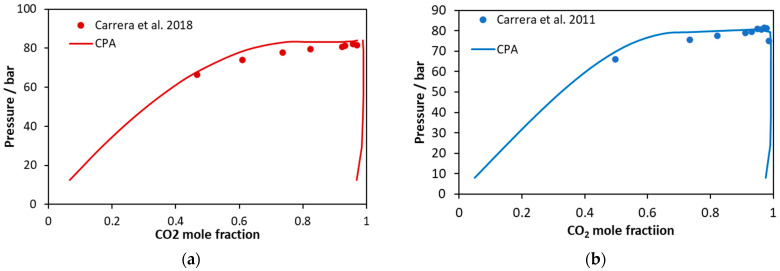
CO_2_ mole fractions in liquid and vapor phases of (**a**) CO_2_–glycerol–methanol and (**b**) CO_2_–glycerol–ethanol ternary systems containing alcohol to glycerol ratio equal to 113. Experimental data [59,60] and CPA predictions using the binary interaction parameters of Table 6.

The obtained results are shown in Figure 9, Figure 10 and Figure 11. Considering the CO_2_–glycerol–methanol system, the model presents an average absolute deviation (in bubble point pressure) from the experimental data shown in Figure 9 [55] equal to 8% and from experimental data shown in Figure 11a [59] equal to 4%. As shown in Figure 9, deviations increase as the mixtures become richer in CO_2_.

On the other hand, considering the CO_2_–glycerol-ethanol system shown in Figure 10, the model describes in a more accurate way mixtures that are rich in CO_2_. In this case, the average absolute deviation (in bubble point pressure) from the experimental data shown in Figure 10 [56] and Figure 11b [60] is equal to 14% and 2%, respectively.

**Figure 12:**
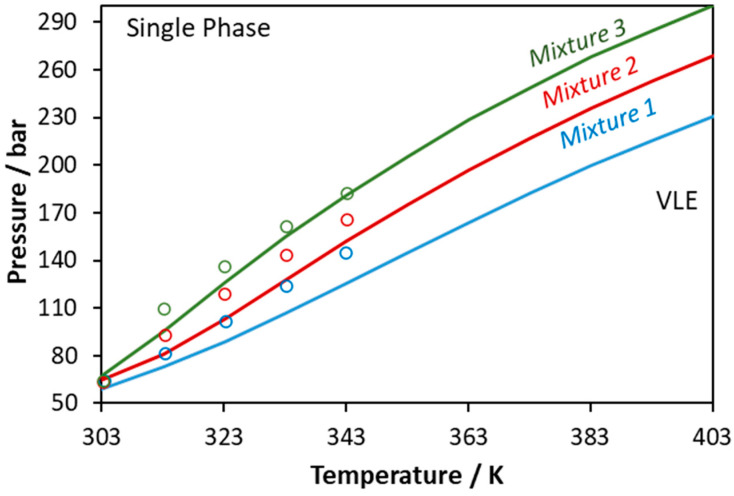
Phase boundaries for CO_2_–methanol–lauric acid ternary system. Experimental data (points, [57]) and CPA predictions using the binary interaction parameters of Table 6 (Mixture 1: x_CO2_ = 0.6675, x_methanol_ = 0.2217, x_lauric acid_ = 0.1108; Mixture 2: x_CO2_ = 0.7492, x_methanol_ = 0.1672, x_lauric acid_ = 0.0836; Mixture 3: x_CO2_ = 0.8248, x_methanol_ = 0.1168, x_lauric acid_ = 0.0584).

Next, CPA was applied to predict the phase behavior of the ternary CO_2_–methanol–lauric acid mixture. Such mixture is reactive since it contains both an alcohol and an organic acid, which renders the accurate measurement and modeling of the phase behavior a demanding task. Similarly to the previous systems, CO_2_ was modeled assuming one positive association site that can only cross-associate with methanol. The obtained results are presented in Figure 12. The model shows an average absolute deviation (in bubble point pressure) from the experimental data shown in Figure 12 [57] equal to 9%. Ferreira et al., who obtained the experimental data, used the Peng–Robinson EoS and in total six binary parameters (two binary parameters per sub-binary system), while two of them (those referring to the methanol–lauric acid mixture) were adjusted to the ternary system’s data [57]. According to their results, the experimental data are satisfactorily correlated.

**Figure 13:**
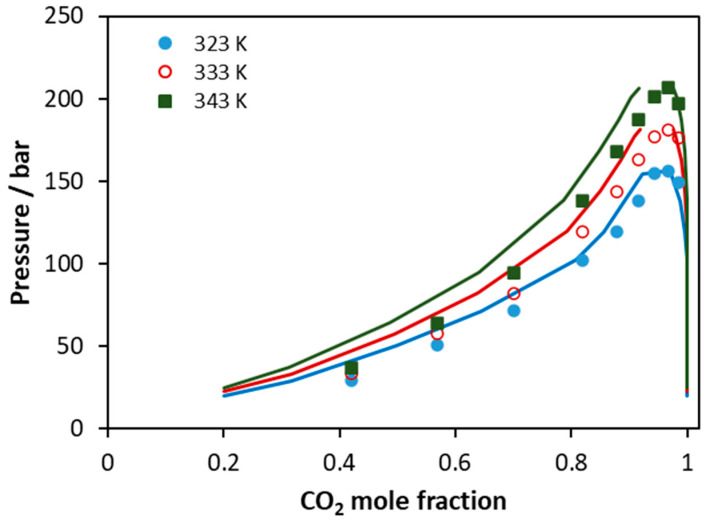
VLE of CO_2_–biodiesel system. Experimental data (points, [58]) and CPA predictions (lines) using the binary interaction parameters of Table 6.

Finally, the CPA EoS was applied to predict the phase behavior of a CO_2_ mixture with a real biodiesel sample (Figure 13). The experimental data from Araujo et al. were used, and biodiesel was considered as a multicomponent mixture of C16:0, C18:0, C18:1, C18:2, and C18:3 methyl esters, according to the analysis mentioned in that study [58]. The CO_2_–methyl ester binary interaction parameters were adopted from Tsivintzelis et al. [7], while, since such compounds have similar molecular structure, the corresponding ester-ester binary interaction parameter was set equal to zero. The overall average absolute deviations of model predictions from experimental data in CO_2_ mole fraction is 8% (7% for 323 K, 8% for 333 K, and 10% for 343 K). It is observed that the deviation increases with temperature; however, the performance of the model is considered rather satisfactory if we take into account that calculations are pure predictions and also the complexity of such multicomponent mixture.

## 4. Overview of Multicomponent Systems Modeled with the CPA EoS

This work completes an extensive work on modeling with the CPA EoS the phase behavior of mixtures relevant to the biodiesel process, which may contain methanol, ethanol, glycerol, fatty acids, esters of fatty acids, glycerides, and CO_2_. In this series of articles (this work and [7,8,30]), pure fluid parameters for such compounds are reported, along with correlations against the molecular weight or the van der Waals volume, which allow the prediction in cases of lack of experimental data. Moreover, the binary parameters for all corresponding binary systems are provided, and in many cases, correlations of such parameters with the van der Waals volume are reported. The capabilities and the limitations of the model are revealed through the prediction of the phase behavior of ternary and multicomponent systems without adjusting any parameter to such experimental data, but only using the sub-binary mixture parameters that were estimated solely from data for binary mixtures. Thus, no “fine-tuning” of those interaction parameters was performed by considering ternary or multicomponent systems data. In this sense, all calculations for ternary or multicomponent mixtures are considered as pure predictions.

In this work and in all our related previous works [7,8,30], we modeled alcohols using the 2B association scheme, water with the 4C association scheme, and glycerol with the 3 × 2B association scheme. In all cases, cross-association interactions among those hydrogen-bonding fluids were taken into consideration. Furthermore, esters of fatty acids, glycerides, and CO_2_ were modeled assuming that their molecules have sites that are only able to cross-associate with other hydrogen-bonding fluids such as alcohols, water, and glycerol. Using one or two binary parameters per binary system, the CPA EoS was able to satisfactorily correlate the phase behavior of binary mixtures. The same approach to account for cross-association interactions was used in ternary or multicomponent mixtures, and it was shown that the model presents a rather satisfactory prediction ability. Numerous ternary or multicomponent mixtures were investigated. Table 7 provides an overview of multicomponent mixtures considered in this work and our previous studies [7,8,30,61] but also in other literature studies with CPA calculations [18,19,20,21,22,23,24,25].

## 5. Conclusions

In this study, the CPA pure fluid parameters for polyethylene glycols were obtained by estimating the trends of such parameters with the molecular weight. Using one temperature-independent binary interaction parameter, the model accurately correlates the solubility of CO_2_ in liquid polyethylene glycols of various molecular weights.

Subsequently, the model was applied to describe the phase behavior of the CO_2_–glycerol system, and it was shown that accounting for cross-association interactions and using two temperature-independent binary parameters, the model accurately describes both phases in equilibrium and especially the vapor phase, which contains a very low amount of the heavy compound, rendering the accurate modeling of such systems difficult. In addition, CPA correlations revealed that the glycerol content of the vapor phase shows a minimum when plotted against pressure, which may be of interest in separation processes, similarly to the CO_2_–water system.

Then, the model was applied to correlate the phase behavior of CO_2_–triglycerides in a wide temperature range in cases for which experimental data are available. Using one temperature-independent parameter, the CPA EoS is able to describe rather satisfactorily the phase behavior, including the very low vapor phase content of the heavy compound. In order to obtain reasonable compositions for the vapor phase, relatively increased but still satisfactory deviations for the liquid phase were obtained.

Subsequently, the model was applied to predict the dew points of three ternary systems. Using no optimized parameters to such data, the model presents average absolute deviations in dew point pressures ranging from 6–14%. Finally, the CPA EoS rather satisfactorily predicted the VLE of a multicomponent mixture containing CO_2_ and a real biodiesel sample, showing an average absolute deviation on CO_2_ mole fractions equal to 8.2%.

This work completes a series of studies in modeling the phase behavior of mixtures relevant to the production of biodiesel [7,8,30]. In such studies, all pure fluid and binary parameters of most important systems are presented, and the predicting ability of the model is evaluated. It is shown that the CPA model presents a satisfactory prediction ability for such systems including mixtures of real biodiesel samples.

## Figures and Tables

**Table 1:** Pure fluid parameters (the association scheme is mentioned in parenthesis).

Fluid	Temp. Range (K)	*T_c_* (K)	*a_0_* (L^2^ bar/moL^2^)	*b* (L/moL)	*c_1_*	*ε* (bar L/moL)	*β*	% AAD ^a^ *P^sat^*/*V^molar^*	Ref.
CO_2_	216–274	304.2	3.5079	0.0272	0.7602	-	-		[31]
Methanol	-	512.6	4.0531	0.0310	0.4310	245.91	0.0161	0.6/0.5	[37]
Ethanol	-	513.9	8.6716	0.0491	0.7369	215.32	0.0080	1.3/0.3	[37]
Glycerol (3 × 2B)	340–680	850.0	11.80	0.0696	0.90	207.85	0.0133	0.7/1.8	[7]
Ethylene glycol (4C)	290–576	720.0	13.259	0.0521	0.9245	143.37	0.0188	2.6/1.5	This work
Diethylene glycol (4C)	324–596	744.6	24.029	0.0922	1.2409	143.37	0.0188	4.2/1.3	This work
Triethylene glycol (4C)	347–616	769.5	38.485	0.1323	1.2254	143.37	0.0188	4.1/1.5	This work
Tetraethylene glycol (4C)	376–636	795.0	53.936	0.1751	1.4228	143.37	0.0188	0.6/2.0	This work
Lauric acid (1A)	334–668	743.0	72.958	0.2270	1.6113	236.08	7.75 × 10^−4^	0.7/2.1	[7]
Methyl palmitate	304–609	762.2	105.049	0.3155	1.5496	-	-	0.4/3.1	[7]
Methyl stearate	312–622	781.1	122.585	0.3518	1.5966	-	-	0.8/3.1	[7]
Methyl oleate	305–611	764.0	115.416	0.3324	1.6709	-	-	2.1/3.2	[7]
Methyl linoleate	278–363	795.3	105.250	0.3189	1.7639	-	-	1.9/1.1	[7]
Methyl linolenate	340–435	797.2	105.310	0.3117	1.7642	-	-	2.1/1.1	[7]
Tributyrin	318–364	658.7	135.39	0.3005	1.0563	-	-	0.7/-	[7]
Tricaprylin	401–452	793.4	239.40	0.5202	1.5765	-	-	2.4/-	[7]
Trilaurin	461–517	869.8	369.12	0.7400	1.8191	-	-	0.9/-	[7]
Trimyristin	489–548	898.6	444.95	0.8498	1.9035	-	-	1.1/-	[7]
Triolein	-	977.9	555.89	1.0506	2.1070	-	-	(prediction)	[7]

^a^$\%AAD=100\cdot \left|X_{exp}-X_{calc}\right|/X_{exp}$, where *X* is the property of interest, while *exp* and *calc* stand for experimental and calculated, respectively.

**Table 2:** Pure fluid parameters for polyethylene glycols; the average molecular weight is shown in parenthesis.

^a^ Fluid	*T_c_* (K)	^b^*a*_0_ (L^2^ bar/moL^2^)	^c^*a*_0_ (L^2^ bar/moL^2^)	*b* (L/moL)	*c*_1_	*ε* (bar L/moL)	*β*
PEG (150)	766	38.023	39.197	0.1332	1.2993	143.37	0.0188
PEG (200)	812	56.337	54.688	0.1797	1.4135	143.37	0.0188
PEG (300)	961	102.004	85.670	0.2726	1.5744	143.37	0.0188
PEG (400)	1186	159.723	116.652	0.3654	1.6885	143.37	0.0188
PEG (600)	1860	311.315	178.616	0.5512	1.8494	143.37	0.0188

^a^ The 4C association scheme was used; ^b^ obtained from polynomial correlation; ^c^ obtained from linear correlation.

**Table 3:** Binary interaction parameters (*k_ij_*) and deviations from experimental data for CO_2_–polyethylene glycols (the average molecular weight is shown in parenthesis).

System	Temp. Range (K)	*k_ij_*	% AAD in Pressure	Exp. Data
CO_2_–PEG (150)	303–333	0.0420	3.5	[38]
CO_2_–PEG (200)	303–333	0.0195	4.0	[38]
CO_2_–PEG (300)	303–333	−0.0200	4.3	[38]
CO_2_–PEG (400)	303–333	−0.0617	3.8	[38]

**Table 4:** Binary parameters and CPA deviations from experimental data [39,40,41,42] (296–393 K) for CO_2_ (1)–glycerol (2).

Approach	*k_ij_*	*β_cross_*	Liquid Phase AAD in *x*_1_	Vapor Phase AAD in *y*_2_
CO_2_ inert	0.1643	-	15.09	98.5
CO_2_ with one association site	0.3084	0.0979	20.0	40.2

**Table 5:** Binary parameters and CPA deviations from experimental data CO_2_ (1)–glycerides (2).

System	Temp. Range (K)	*k_ij_*	Liquid Phase AAD in *x*_1_	Vapor Phase AAD in *y*_2_	Exp. Data
CO_2_–Tricaprylin	313–393	0.0522	8.9	35.8	[43]
CO_2_–Tributyrin	278–368	−0.0165	5.3	-	[44,45]
CO_2_–Trilaurin	308–353	0.0708	11.3	63.7	[46,47,48,49]
CO_2_–Trimyristin	308–328	0.0905	-	56.3	[46,48]
CO_2_–Triolein	308–363	0.0585	8.9	139	[50,51,52,53,54]

**Table 6:** Binary parameters used in calculations for ternary or multicomponent systems.

Sub-Binary Mixture	*k_ij_*	ε_cross_ (bar L/mol)	*β_cross_*	Reference
CO_2_–Methanol	0.0479	123.8 (exp.)	0.0196	[31]
CO_2_–Ethanol	0.1132	123.8 (exp.)	0.0320	[31]
CO_2_–Glycerol	0.3084	mCR1 ^a^	0.0979	This work, see Table 4
CO_2_–Lauric acid	0.0380	-	-	[7]
CO_2_–Methyl linoleate	0.0480	-	-	[7]
CO_2_–Methyl oleate	0.0566	-	-	[7]
CO_2_–Methyl linolenate	0.3000	-	-	[7]
CO_2_–Methyl palmitate	0.0653	-	-	[7]
CO_2_–Methyl stearate	0.0715	-	-	[7]
Glycerol–Methanol	0.0836	CR1 ^a^	CR1 ^a^	[7]
Glycerol–Ethanol	0.0226	CR1 ^a^	CR1 ^a^	[7]
Lauric acid–Methanol	−0.0181	mCR1 ^a^	0.1028 ^b^	[7]

^a^ For combining rule 1 (CR1) and modified combining rule 1 (mCR-1), see ref. [31]. ^b^ Parameters adopted from methanol–methyl laurate in absence of parameters for the particular system.

**Table 7:** Overview of multicomponent systems modeled with the CPA EoS.

System	Reference
CO_2_–methyl myristate–methyl palmitate	[8]
CO_2_–methyl oleate–methyl linoleate	[8]
CO_2_–methanol–lauric acid	This work
CO_2_–glycerol–methanol	This work
CO_2_–glycerol–ethanol	This work
CO_2_–water–methanol	[16,29,61]
CO_2_–water–ethanol	[29]
CO_2_–various biodiesel samples	This work, [8]
Methanol–glycerol–methyl oleate	[19,20,24,30]
Methanol–glycerol–methyl ricinoleate	[20]
Methanol–glycerol–methyl oleate–hexane	[20,30]
Ethanol–glycerol–ethyl laurate	[7,24]
Ethanol–glycerol–ethyl oleate	[25,30]
Ethanol–glycerol–ethyl linoleate	[25,30]
Ethanol–glycerol–ethyl palmitate	[25,30]
Ethanol–glycerol–ethyl myristate	[20]
Ethanol–glycerol–methyl stearate	[20]
Ethanol–water–ethyl laurate	[22,24]
Ethanol–water–ethyl myristate	[22]
Various biodiesel samples–CO_2_	This work, [8]
Various biodiesel samples–water	[18,30]
Various biodiesel samples–glycerol–methanol	[7]
Various biodiesel samples–glycerol–ethanol	[23]

## Data Availability

Not applicable.

## Supplementary Materials

The following supporting information can be downloaded at: https://www.mdpi.com/article/10.3390/molecules27185785/s1, Figure S1: CO_2_–glycerol VLE. Glycerol mole fraction in the vapor phase. Experimental data and CPA correlations using the interaction parameters of Table 4 of the main article; Figure S2: CO_2_–glycerol VLE. CO_2_ mole fraction in the liquid phase. Experimental data [41,42] and CPA correlations using the interaction parameters of Table 4 of the main article; Figure S3: CO_2_–glycerol VLE. CO_2_ mole fraction in the liquid phase. Experimental data [39,41,42] and CPA correlations using the interaction parameters of Table 4 of the main article; Figure S4: CO_2_–glycerol VLE. CO_2_ mole fraction in the liquid phase. Experimental data [42] and CPA correlations using the interaction parameters of Table 4 of the main article; Figure S5: CO_2_–glycerol VLE. CO_2_ mole fraction in the liquid phase. Experimental data [42] and CPA correlations using the interaction parameters of Table 4 of the main article; Figure S6: CO_2_–glycerol VLE. CO_2_ mole fraction in the liquid phase. Experimental data [39] and CPA correlations using the interaction parameters of Table 4 of the main article; Figure S7: CO_2_–tricaprilyn VLE at 313 K. Experimental data [43,44] and CPA correlations using the binary parameter of Table 5 of the main article; Figure S8: CO_2_–tricaprilyn VLE at 393 K. Experimental data [43] and CPA correlations using the binary parameter of Table 5 of the main article; Figure S9: CO_2_–tributyrin VLE at 288 K. Experimental data [44,45] and CPA correlations using the binary parameter of Table 5 of the main article; Figure S10: CO_2_–tributyrin VLE at 303 K. Experimental data [44,45] and CPA correlations using the binary parameter of Table 5 of the main article; Figure S11: CO_2_–tributyrin VLE at 338 K. data [44,45] and CPA correlations using the binary parameter of Table 5 of the main article; Figure S12: CO_2_–trilaurin VLE and LLE at 308 K. Experimental data [48,49] and CPA correlations using the binary parameter of Table 5 of the main article; Figure S13: CO_2_–trilaurin VLE at 328 K. Experimental data [48] and CPA correlations using the binary parameter of Table 5 of the main article; Figure S14: CO_2_–trilaurin VLE at 353 K. Experimental data [47] and CPA correlations using the binary parameter of Table 5 of the main article; Figure S15: CO_2_–trimyristin VLE at 308 K. Experimental data [48] and CPA correlations using the binary parameter of Table 5 of the main article; Figure S16: CO_2_–trimyristin VLE at 328 K. Experimental data [48] and CPA correlations using the binary parameter of Table 5 of the main article; Figure S17: CO_2_–triolein VLE at 313 K (vapor phase compositions). Experimental data [53,54] and CPA correlations using the binary parameter of Table 5 of the main article; Figure S18: CO_2_–triolein VLE at 323 K (vapor phase compositions). Experimental data [50,53,54] and CPA correlations using the binary parameter of Table 5 of the main article; Figure S19: CO_2_–triolein VLE at 333 K. Experimental data [51,52,53] and CPA correlations using the binary parameter of Table 5 of the main article; Figure S20. CO_2_–triolein VLE at 343 K. Experimental data [52,53] and CPA correlations using the binary parameter of Table 5 of the main article.

## References

[1] Temelli F. (2009). Perspectives on supercritical fluid processing of fats and oils. J. Supercrit. Fluids.

[2] Kultys E., Kurek M.A. (2022). Green Extraction of Carotenoids from Fruit and Vegetable Byproducts: A Review. Molecules.

[3] Baranauskienė R., Venskutonis P.R. (2022). Supercritical CO_2_ Extraction of *Narcissus poeticus* L. Flowers for the Isolation of Volatile Fragrance Compounds. Molecules.

[4] Macaira J., Santana A., Costa A., Ramirez E., Larroyoz M.A. (2014). Process intensification using CO2 as a cosolvent under super-critical conditions applied to design of biodiesel production. Ind. Eng. Chem. Res..

[5] Kenarsari S.D., Yang D., Jiang G., Zhang S., Wang J., Russell A.G., Wei Q., Fan M. (2013). Review of recent advances in carbon dioxide separation and capture. RSC Adv..

[6] Tsivintzelis I., Kontogeorgis G.M., Panayiotou C. (2017). Dimerization of Carboxylic Acids: An Equation of State Approach. J. Phys. Chem. B.

[7] Tsivintzelis I., Ali S., Kontogeorgis G.M. (2016). Modeling systems relevant to the biodiesel production using the CPA equation of state. Fluid Phase Equilibria.

[8] Tsivintzelis I., Ali S., Kontogeorgis G.M. (2019). Modeling systems relevant to the biodiesel production using the CPA equation of state. Part 2. Systems with supercritical CO2. Fluid Phase Equilibria.

[9] Kontogeorgis G.M., Voutsas E.C., Yakoumis I.V., Tassios D.P. (1996). An Equation of State for Associating Fluids. Ind. Eng. Chem. Res..

[10] Kontogeorgis G.M., Michelsen M.L., Folas G.K., Derawi S., von Solms N., Stenby E.H. (2006). Ten years with the CPA (Cubic-Plus-Association) equation of state. Part 1. Pure compounds and self-associating systems. Ind. Eng. Chem. Res..

[11] Kontogeorgis G.M., Michelsen M.L., Folas G.K., Derawi S., von Solms N., Stenby E.H. (2006). Ten years with the CPA (Cubic-Plus-Association) equation of state. Part 2. Cross-associating and multicomponent systems. Ind. Eng. Chem. Res..

[12] Derawi S.O., Kontogeorgis G.M., Michelsen M.L., Stenby E.H. (2003). Extension of the Cubic-Plus-Association Equation of State to Glycol−Water Cross-Associating Systems. Ind. Eng. Chem. Res..

[13] Breil M.P., Kontogeorgis G.M. (2009). Thermodynamics of Triethylene Glycol and Tetraethylene Glycol Containing Systems De-scribed by the Cubic-Plus-Association Equation of State. Ind. Eng. Chem. Res..

[14] Arya A., Maribo-Mogensen B., Tsivintzelis I., Kontogeorgis G. (2014). Process Design of Industrial Triethylene Glycol Processes Using the Cubic-Plus-Association (CPA) Equation of State. Ind. Eng. Chem. Res..

[15] Tsivintzelis I., Kontogeorgis G.M. (2016). Modelling phase equilibria for acid gas mixtures using the CPA equation of state. Part VI. Multicomponent mixtures with glycols relevant to oil and gas and to liquid or supercritical CO_2_ transport applications. J. Chem. Thermodyn..

[16] Tsivintzelis I., Bjørner M.G., Kontogeorgis G.M. (2018). Recent advances with association models for practical applications. Mol. Phys..

[17] Oliveira M., Marrucho I., Coutinho J., Queimada A. (2008). Surface tension of chain molecules through a combination of the gradient theory with the CPA EoS. Fluid Phase Equilibria.

[18] Oliveira M.B., Varanda F.R., Marrucho I., Queimada A.J., Coutinho J. (2008). Prediction of Water Solubility in Biodiesel with the CPA Equation of State. Ind. Eng. Chem. Res..

[19] Oliveira M.B., Teles A.R.R., Queimada A.J., Coutinho J.A.P. (2009). Phase equilibria of glycerol containing systems and their de-scription with the Cubic-Plus-Association (CPA) equation of state. Fluid Phase Equilibria.

[20] Oliveira M.B., Queimada A.J., Coutinho J.A. (2009). Modeling of Biodiesel Multicomponent Systems with the Cubic-Plus-Association (CPA) Equation of State. Ind. Eng. Chem. Res..

[21] Oliveira M.B., Queimada A.J., Kontogeorgisc G.M., Coutinho J.A.P. (2011). Evaluation of the CO_2_ behavior in binary mixtures with alkanes, alcohols, acids and esters using the Cubic-Plus-Association equation of state. J. Supercrit. Fluids.

[22] Follegatti-Romero L.A., Lanza M., Batista F.R.M., Batista E.A.C., Oliveira M.B., Coutinho J.A.P., Meirelles J.A. (2010). Liquid-Liquid equilibrium for ternary systems containing ethyl esters, anhydrous ethanol and water at 298.15, 313.15 and 333.15 K. Ind. Eng. Chem. Res..

[23] Oliveira M.B., Barbedo S., Soletti J.I., Carvalho S.H., Queimada A.J., Coutinho J.A. (2011). Liquid–liquid equilibria for the canola oil biodiesel + ethanol + glycerol system. Fuel.

[24] Oliveira M.B., Ribeiro V., Quemada A.J., Coutinho J.A.P. (2011). Modeling phase equilibria relevant to biodiesel production: A comparison of GE models, Cubic EoS, EoS-GE and association EoS. Ind. Eng. Chem. Res..

[25] Follegatti-Romero L.A., Oliveira M.B., Batista F.R.M., Batista E.A.C., Coutinho J.A.P., Meirelles J.A.A. (2012). Liquid-Liquid equilibrium for ternary systems containing ethyl esters, ethanol and glycerol at 323.15 and 353.15 K. Fuel.

[26] Li Y., Qiao Z., Sun S., Zhang T. (2020). Thermodynamic modeling of CO_2_ solubility in saline water using NVT flash with the cubic-plus-association equation of state. Fluid Phase Equilib..

[27] Tsivintzelis I., Shahid A., Kontogeorgis G.M. (2014). Modeling phase equilibria for acid gas mixtures using the CPA equation of state. Part 3. Applications relevant to liquid or supercritical CO_2_ transport. J. Chem. Eng. Data.

[28] Tsivintzelis I., Ali S., Kontogeorgis G.M. (2015). Modeling phase equilibria for acid gas mixtures using the CPA equation of state. Part IV. Applications to mixtures of CO_2_ with alkanes. Fluid Phase Equilibria.

[29] Tsivintzelis I., Kontogeorgis G.M. (2015). Modeling Phase Equilibria for Acid Gas Mixtures using the CPA Equation of State. Part V. Multicomponent mixtures of containing CO_2_ and alcohols. J. Supercrit. Fluids.

[30] Tsivintzelis I., Karakatsani E., Kontogeorgis G.M. (2020). Costa Tsonopoulos—his legacy and some personal reflections on cubic equations of state and beyond. Fluid Phase Equilibria.

[31] Tsivintzelis I., Kontogeorgis G.M., Michelsen M.L., Stenby E.H. (2011). Modeling phase equilibria for acid gas mixtures using the CPA equation of state. Part II: Binary mixtures with CO_2_. Fluid Phase Equilibria.

[32] DIPPR 801 Thermophysical Property Database and DIADEM Predictive Proffesional 2011 Version 5.0.1.

[33] Kontogeorgis G.M., Folas G.K. (2010). Thermodynamic models for industrial applications. From Classical and Advanced Mixing Rules to Association Theories.

[34] Stefanis E., Constantinou L., Tsivintzelis I., Panayiotou C. (2005). New group-contribution method for predicting temperature-dependent properties of pure organic compounds. Int. J. Thermophys..

[35] Papaioannou V., Adjiman C.S., Jackson G., Galindo A., Adjiman C.S., Galindo A. (2011). Group contribution methodologies for the prediction of thermodynamic properties and phase behavior in mixtures. Process Systems Engineering: Molecular Systems Engineering.

[36] Poling B.E., Prausnitz J.M., O’Connel J.P. (2001). The Properties of Gases and Liquids.

[37] Folas G.K., Kontogeorgis G.M., Michelsen A.M.L., Stenby E.H. (2006). Application of the Cubic-Plus-Association Equation of State to Mixtures with Polar Chemicals and High Pressures. Ind. Eng. Chem. Res..

[38] Li J., Ye Y., Chen L., Qi Z. (2012). Solubilities of CO2 in Poly(ethylene glycols) from (303.15 to 333.15) K. J. Chem. Eng. Data.

[39] Nunes A.V., Carrera G.V., Najdanovic-Visak V., da Ponte M.N. (2013). Solubility of CO_2_ in glycerol at high pressures. Fluid Phase Equilibria.

[40] Sovová H., Jez J., Khachaturyan M. (1997). Solubility of squalane, dinonyl phthalate and glycerol in supercritical CO_2_. Fluid Phase Equilibria.

[41] Ostonen A., Sapei E., Uusi-Kyyny P., Klemela A., Alopaeus V. (2014). Measurements and modeling of CO_2_ solubility in1,8-diazabicyclo-[5.4.0]-undec-7-ene-Glycerol solutions. Fluid Phase Equilibria.

[42] Décultot M., Ledoux A., Fournier-Salaün M.-C., Estel L. (2019). Solubility of CO_2_ in methanol, ethanol, 1,2-propanediol and glycerol from 283.15 K to 373.15 K and up to 6.0 MPa. J. Chem. Thermodyn..

[43] Borch-Jensen C., Mollerup J. (1997). Phase equilibria of carbon dioxide and tricaprylin. J. Supercrit. Fluids.

[44] Florusse L., Fornari T., Bottini S., Peters C. (2004). Phase behavior of carbon dioxide—low-molecular weight triglycerides binary systems: Measurements and thermodynamic modeling. J. Supercrit. Fluids.

[45] Howlader S., Venkatesan S., Goel H., Huda M., French W.T., Rai N. (2018). Solubility of CO_2_ in triglycerides using Monte Carlo simulations. Fluid Phase Equilibria.

[46] Bamberger T., Erickson J.C., Cooney C.L., Kumar S.K. (1988). Measurement and model prediction of solubilities of pure fatty acids, pure triglycerides, and mixtures of triglycerides in supercritical carbon dioxide. J. Chem. Eng. Data.

[47] Bharath R., Yamane S., Inomata H., Adschiri T., Arai K. (1993). Phase equilibria of supercritical CO_2_-fatty oil component binary systems. Fluid Phase Equilibria.

[48] Pearce D.L. (1990). Solubility of Triglycerides in Supercritical Carbon Dioxide. Ph.D. Thesis.

[49] Ashour I., Hammam H. (1993). Equilibrium Solubility of Pure Mono-, Di-, and Trilaurin in Supercritical Carbon Dioxide-Experimental Measurements and Model Prediction. J. Supercrit. Fluids.

[50] Nilsson W.B., Gauglitz E.J., Hudson J.K. (1991). Solubilities of Methyl Oleate, Oleic Acid, Oleyl Glycerols, Glycerol Mixtures in Supercritical Carbon Dioxide. JAOCS.

[51] Weber W., Petkov S., Brunner G. (1999). Vapour–liquid-equilibria and calculations using the Redlich–Kwong-Aspen-equation of state for tristearin, tripalmitin, and triolein in CO_2_ and propane. Fluid Phase Equilibria.

[52] Perko T., Knez Z., Škerget M. (2012). Phase Equilibria of Glycerol Tristearate and Glycerol Trioleate in Carbon Dioxide and Sulfur Hexafluoride. J. Chem. Eng. Data.

[53] Chrastil J. (1982). Solubility of solids and liquids in supercritical gases. J. Phys. Chem..

[54] Ribeiro M.A., Bernardo-Gil M.G. (1995). Solubilities of Triolein in Supercritical CO_2_. J. Chem. Eng. Data.

[55] Ferreira-Pinto L., Ndiaye P., Ramos L.P., Corazza M.L. (2011). Phase equilibrium data of the system CO_2_+glycerol+methanol at high pressures. J. Supercrit. Fluids.

[56] Araújo O.A., Ndiaye P.M., Ramos L.P., Corazza M.L. (2011). Phase behavior measurement for the system CO_2_+glycerol+ethanol at high pressures. J. Supercrit. Fluids.

[57] Ferreira F.M., Ramos L.P., Ndiaye P.M., Corazza M.L. (2011). Phase behavior of (CO_2_+methanol+lauric acid) system. J. Chem. Thermodyn..

[58] Araújo O.A., Silva F.R., Ramos L.P., Lenzi M.K., Ndiaye P.M., Corazza M.L. (2012). Phase behaviour measurements for the system (carbon dioxide + biodiesel + ethanol) at high pressures. J. Chem. Thermodyn..

[59] Carrera G.V., Visak Z.P., Lukasik R.M., Da Ponte M.N. (2018). CO_2_ + Methanol + Glycerol: Multiphase behaviour. J. Supercrit. Fluids.

[60] Carrera G., Visak Z., Bogel-Lukasik R., da Ponte M.N. (2011). VLE of CO_2_+glycerol+(ethanol or 1-propanol or 1-butanol). Fluid Phase Equilibria.

[61] Tsivintzelis I., Musko N.E., Baiker A., Grunwaldt J.-D., Kontogeorgis G.M. (2013). Experimental determination and modeling of the phase behavior for the direct synthesis of dimethyl carbonate from methanol and carbon dioxide. J. Supercrit. Fluids.

