# Supplementary material for: Modeling Binary and Multicomponent Systems Containing Supercritical CO2 with Polyethylene Glycols and Compounds Relevant to the Biodiesel Production
Source: Molecules. 2022 Sep 7;27(18):5785. doi: 10.3390/molecules27185785 (PMC9506037; doi:10.3390/molecules27185785)
Supplement: Supplementary file 1 [file molecules-27-05785-s001.zip › molecules-1878326-SI.pdf]

Supplementary Information File for

Modeling Binary and Multicomponent Systems Containing Supercritical CO_2 with Polyethylene Glycols and Compounds Relevant to the Biodiesel Production

Ioannis Tsivintzelis[1], George Koutsou[1], Georgios M. Kontogeorgis[2]

[1]Aristotle University of Thessaloniki, Department of Chemical Engineering, 54124, Greece
[2]Center for Energy Resources Engineering (CERE), Department of Chemical and Biochemical Engineering, Technical University of Denmark, DK-2800 Kgs. Lyngby, Denmark

**Corresponding author: Ioannis Tsivintzelis: E-mail: tioannis@cheng.auth.gr*

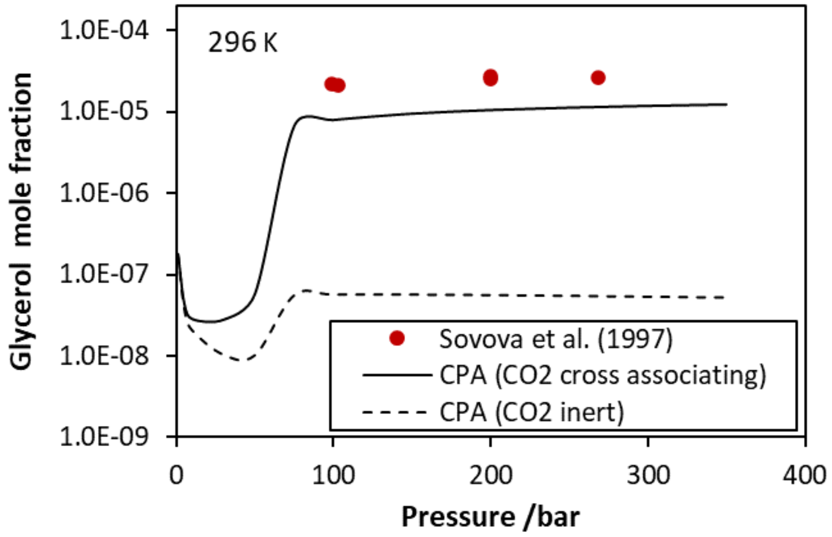

Figure S1. CO_2 - glycerol VLE. Glycerol mole fraction in the vapor phase. Experimental data [40] and CPA correlations using the interaction parameters of Table 4 of the main article.

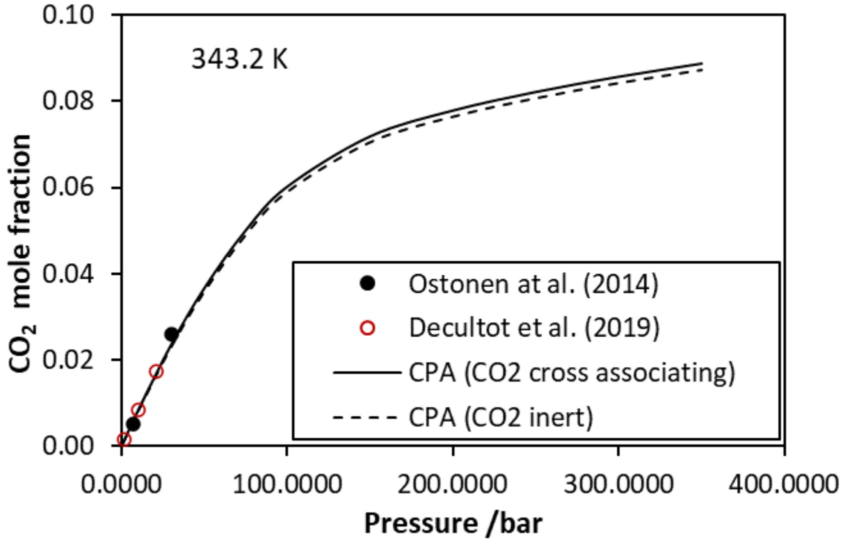

Figure S2. CO_2 - glycerol VLE. CO_2 mole fraction in the liquid phase. Experimental data [41,42] and CPA correlations using the interaction parameters of Table 4 of the main article.

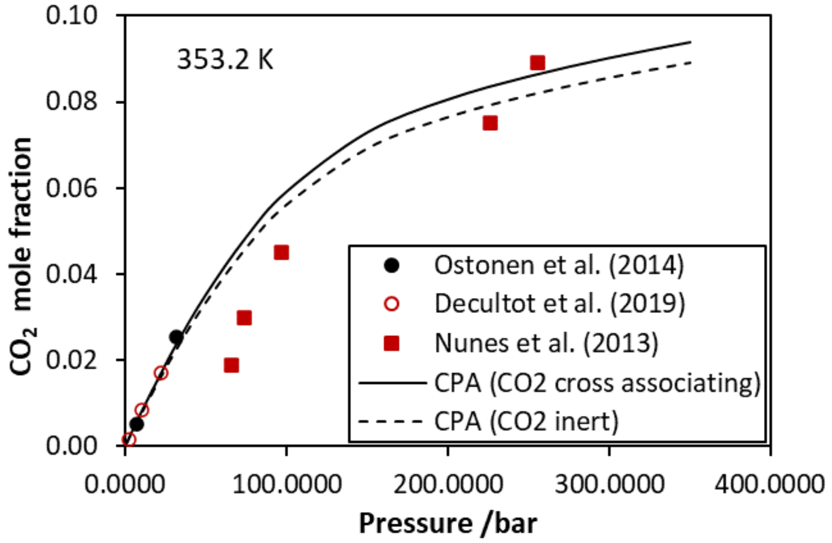

Figure S3. CO_2 - glycerol VLE. CO_2 mole fraction in the liquid phase. Experimental data [39,41,42] and CPA correlations using the interaction parameters of Table 4 of the main article.

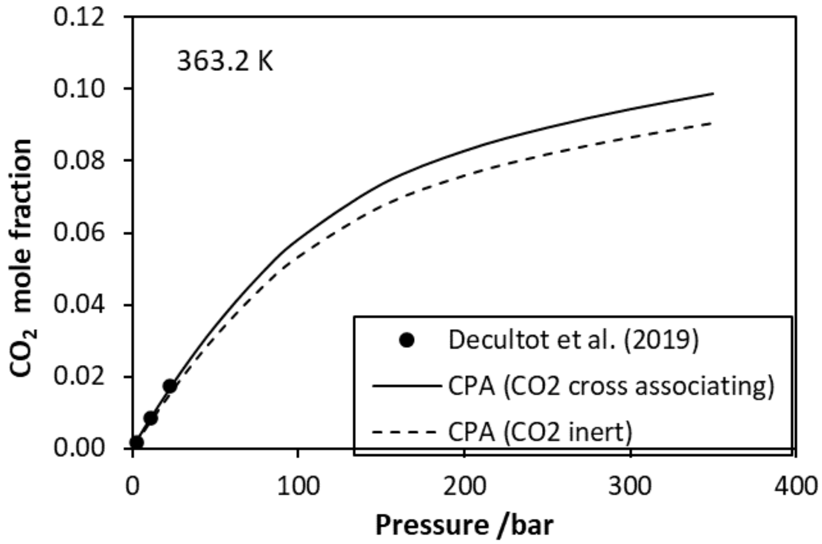

Figure S4. CO_2 - glycerol VLE. CO_2 mole fraction in the liquid phase. Experimental data [42] and CPA correlations using the interaction parameters of Table 4 of the main article.

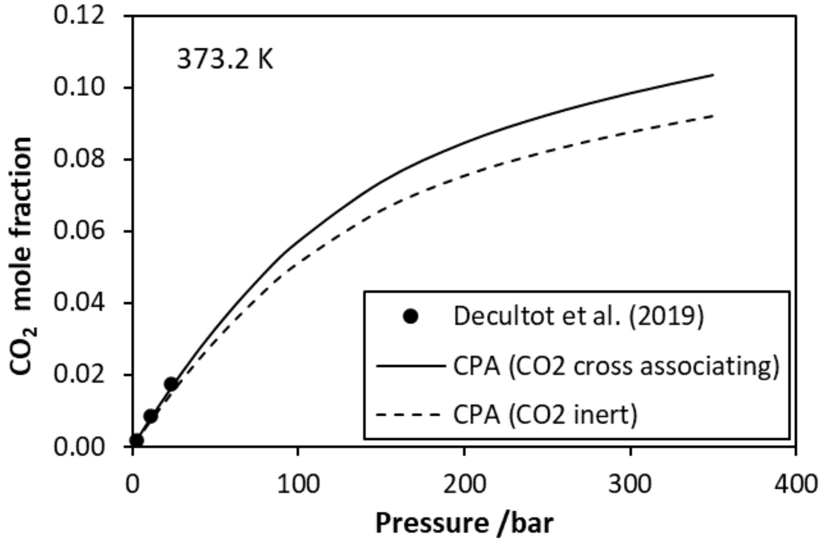

Figure S5. CO_2 - glycerol VLE. CO_2 mole fraction in the liquid phase. Experimental data [42] and CPA correlations using the interaction parameters of Table 4 of the main article.

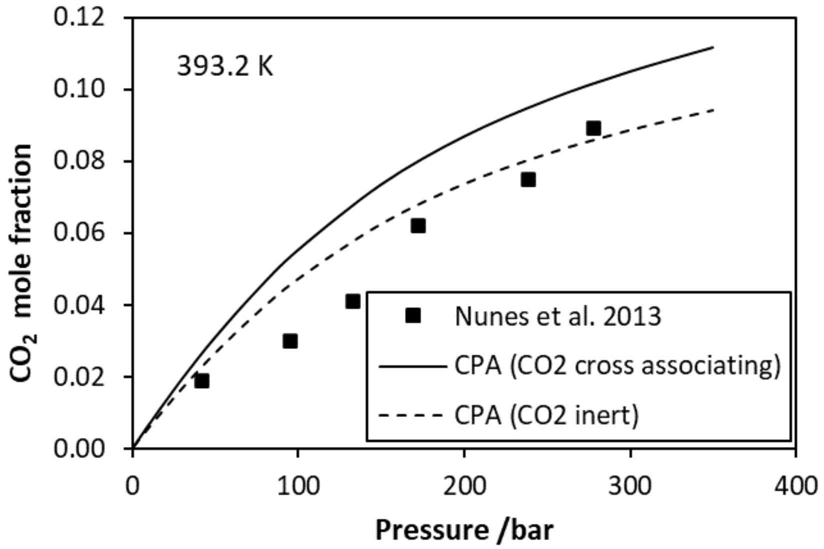

Figure S6. CO_2 - glycerol VLE. CO_2 mole fraction in the liquid phase. Experimental data [39] and CPA correlations using the interaction parameters of Table 4 of the main article.

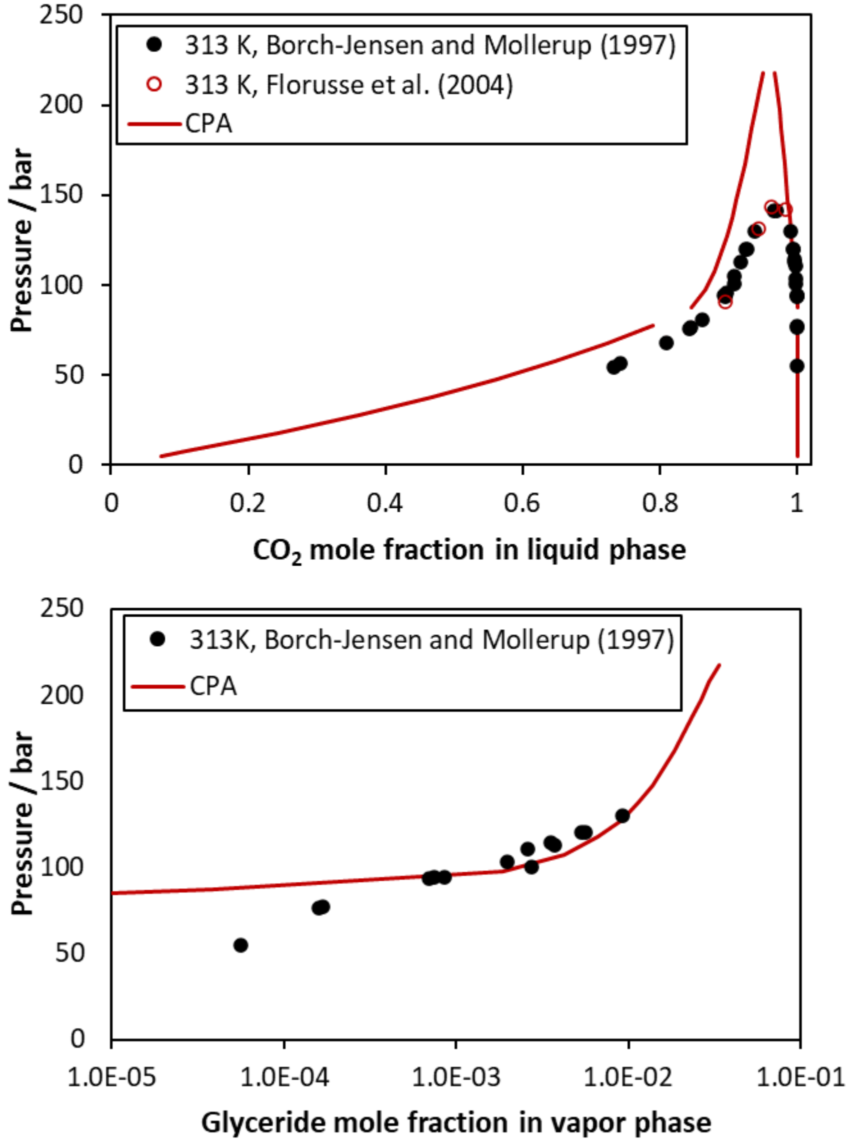

Figure S7. CO_2 - tricaprilyn VLE at 313 K. Experimental data [43,44] and CPA correlations using the binary parameter of Table 5 of the main article.

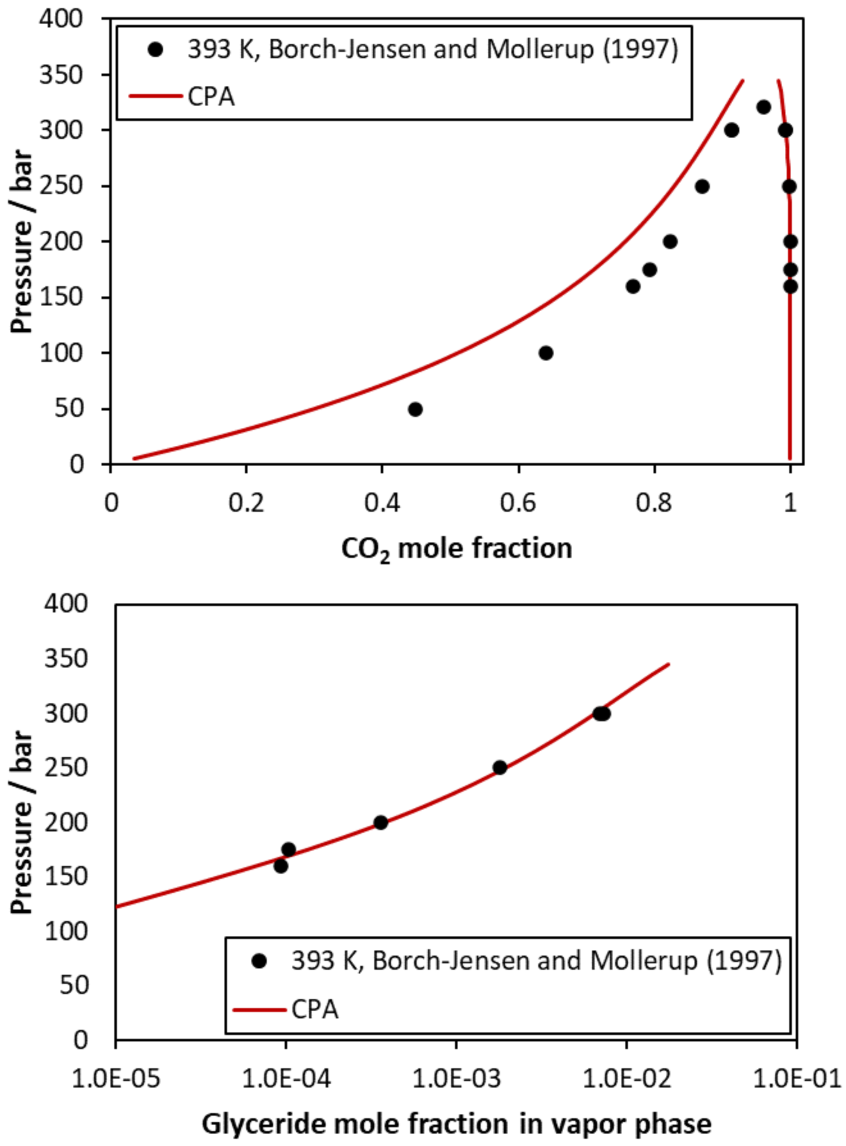

Figure S8. CO_2 - tricaprilyn VLE at 393 K. Experimental data [43] and CPA correlations using the binary parameter of Table 5 of the main article.

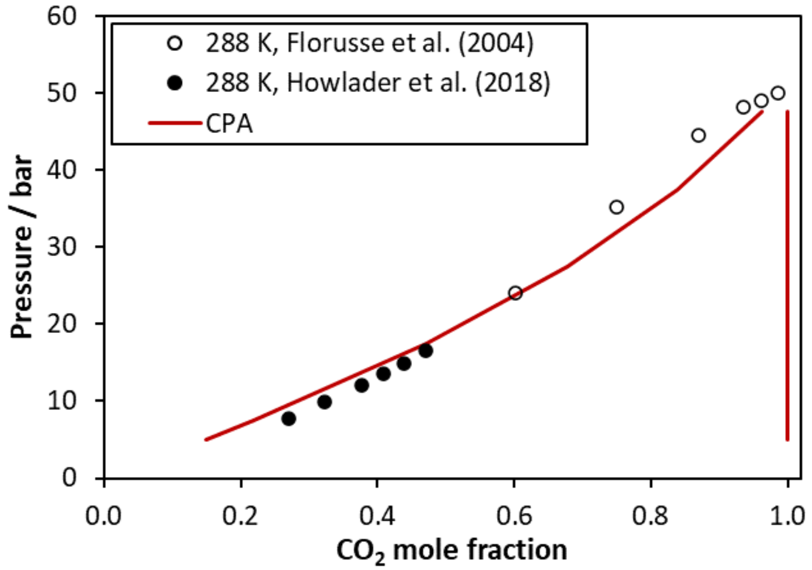

Figure S9. CO_2 - tributyrin VLE at 288 K. Experimental data [44,45] and CPA correlations using the binary parameter of Table 5 of the main article.

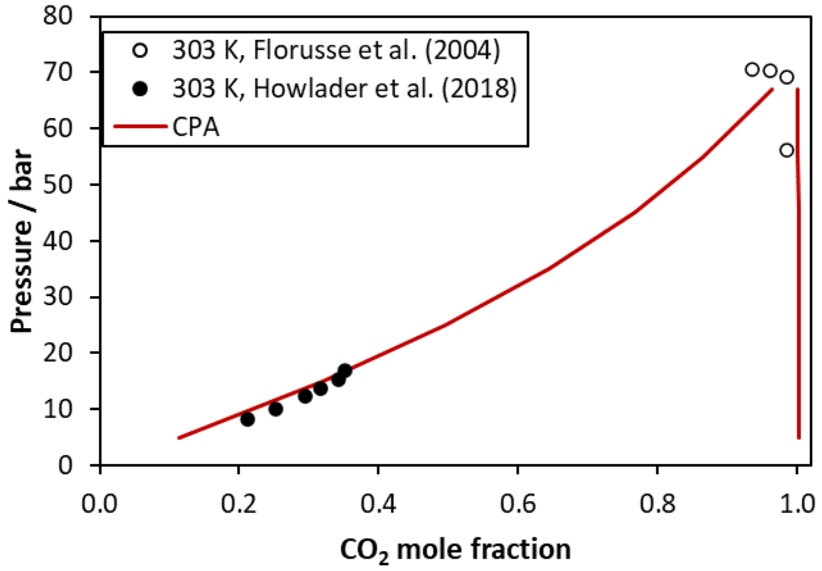

Figure S10. CO_2 - tributyrin VLE at 303 K. Experimental data [44,45] and CPA correlations using the binary parameter of Table 5 of the main article.

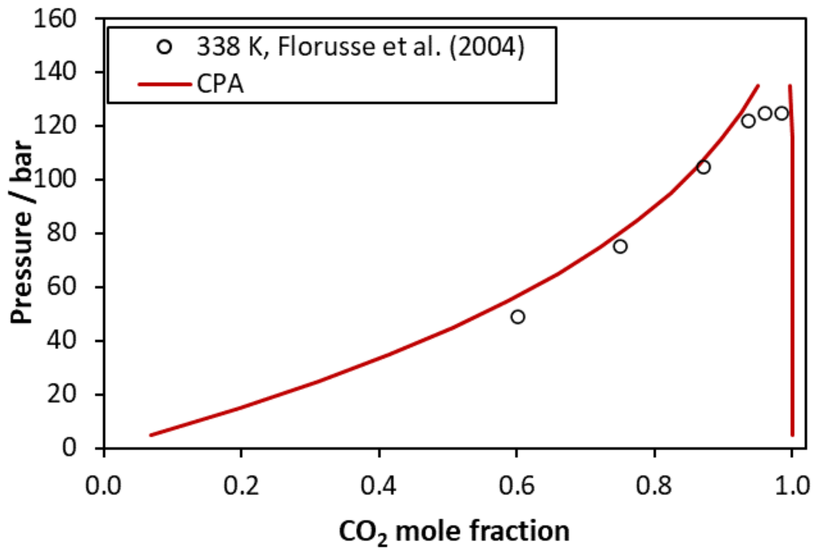

Figure S11. CO_2 - tributyrin VLE at 338 K. data [44,45] and CPA correlations using the binary parameter of Table 5 of the main article.

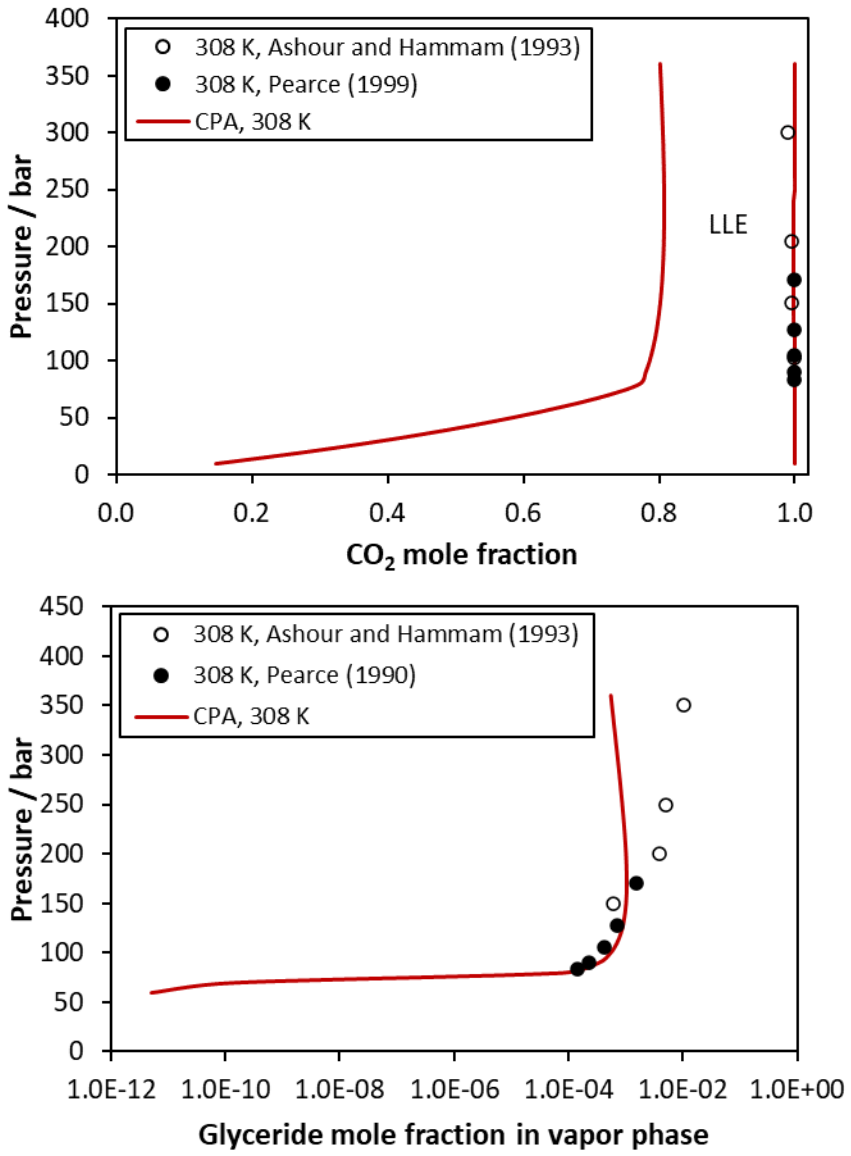

Figure S12. CO_2 - trilaurin VLE and LLE at 308 K. Experimental data [48,49] and CPA correlations using the binary parameter of Table 5 of the main article.

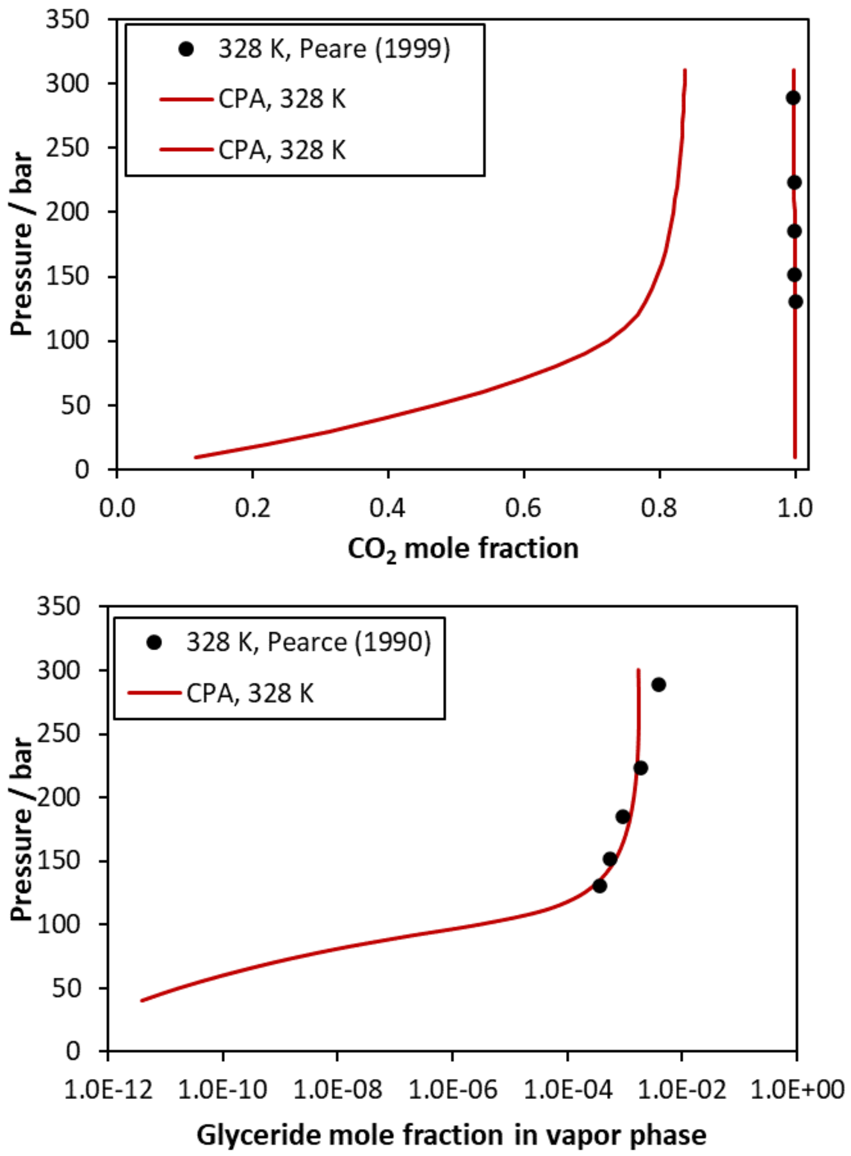

Figure S13. CO_2 - trilaurin VLE at 328 K. Experimental data [48] and CPA correlations using the binary parameter of Table 5 of the main article.

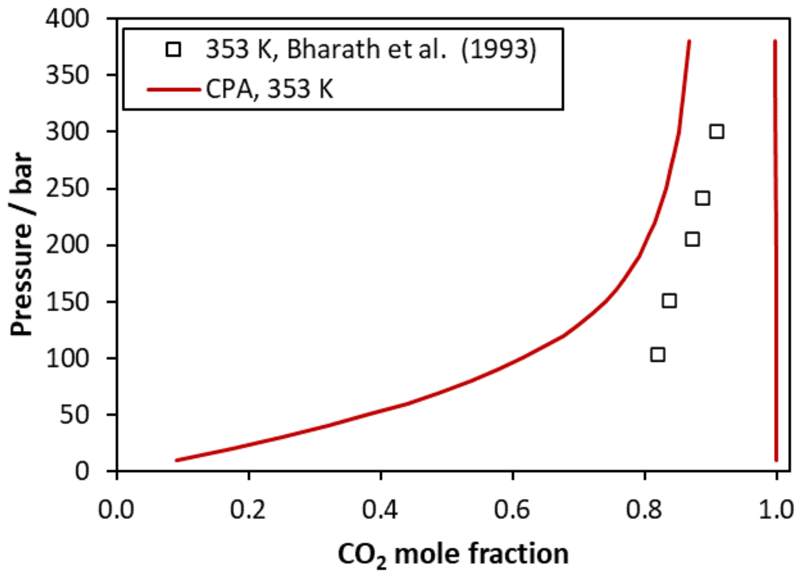

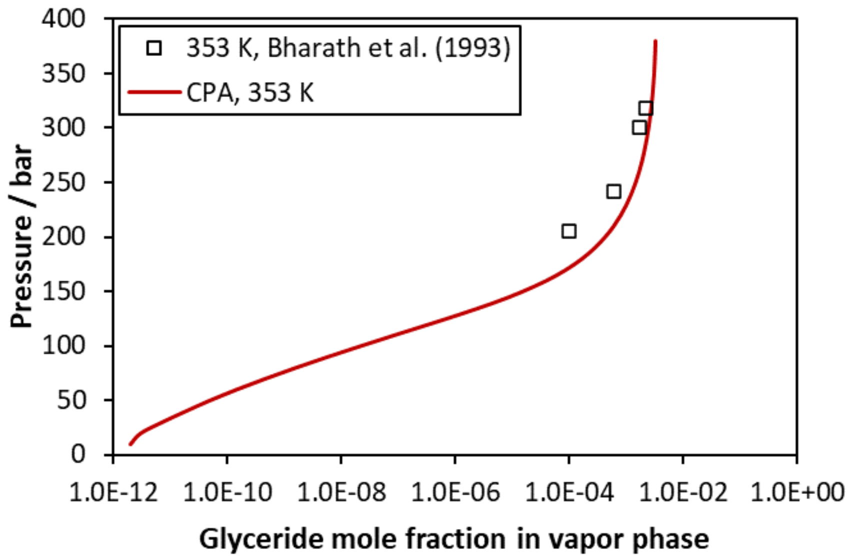

Figure S14. CO_2 - trilaurin VLE at 353 K. Experimental data [47] and CPA correlations using the binary parameter of Table 5 of the main article.

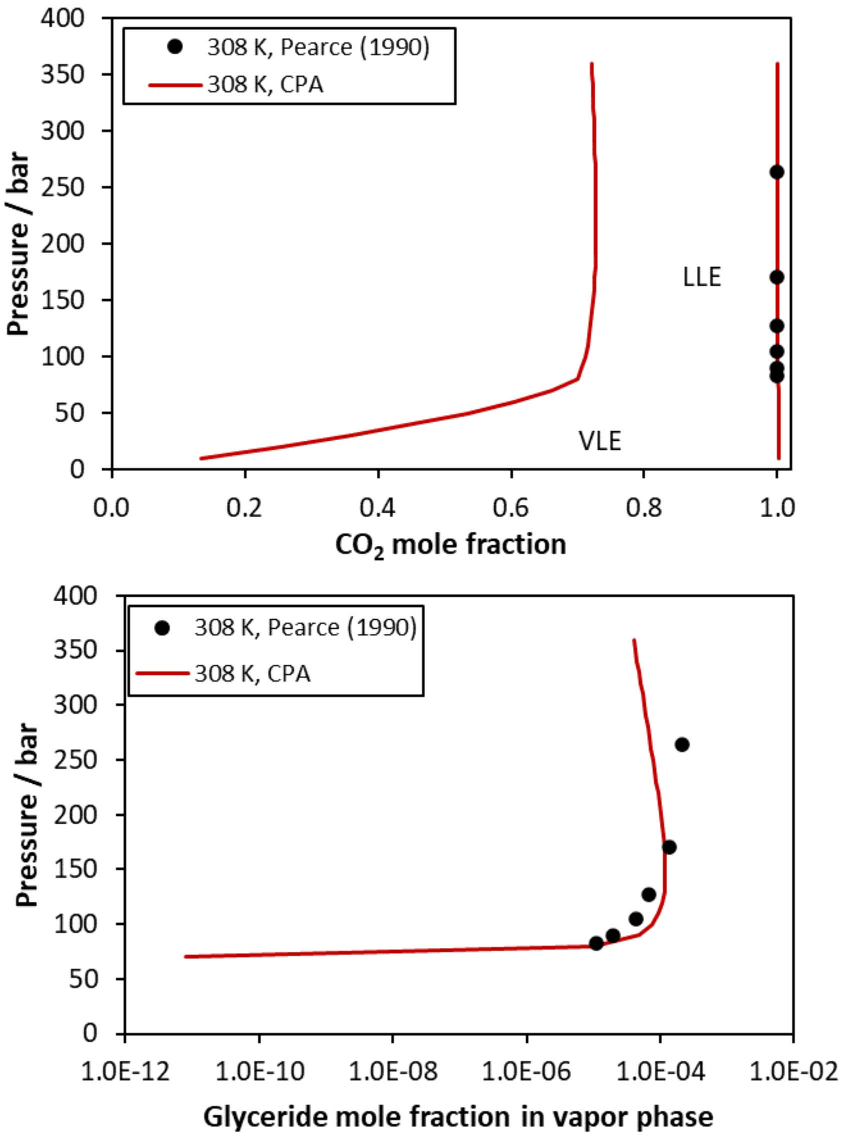

Figure S15. CO_2 – trimyristin VLE at 308 K. Experimental data [48] and CPA correlations using the binary parameter of Table 5 of the main article.

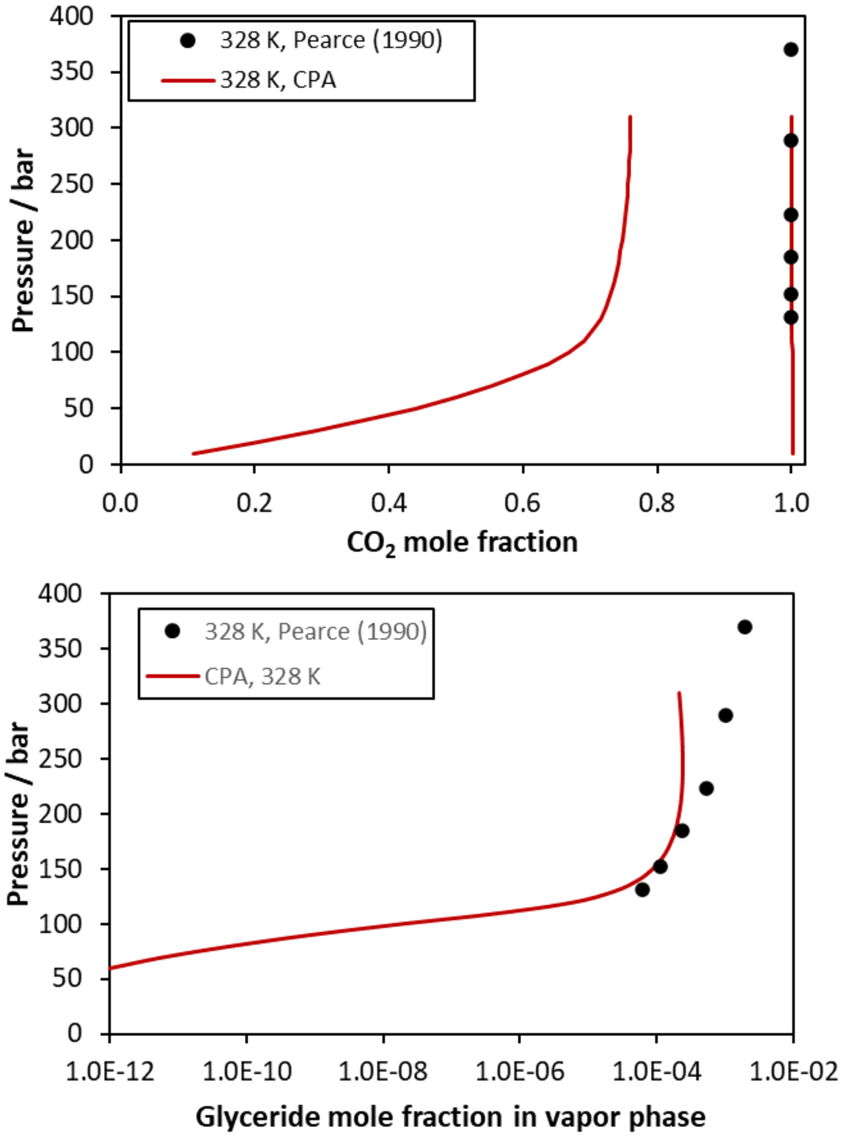

Figure S16. CO_2 – trimyristin VLE at 328 K. Experimental data [48] and CPA correlations using the binary parameter of Table 5 of the main article.

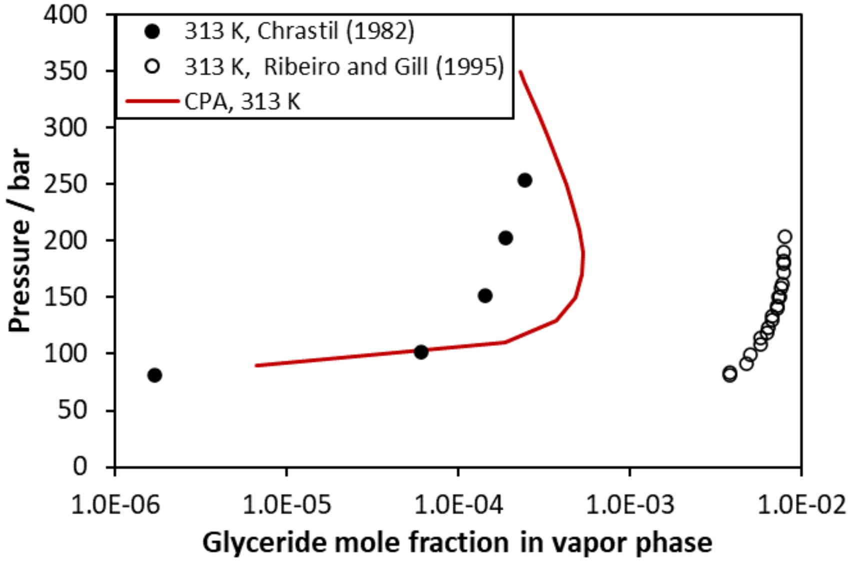

Figure S17. CO_2 – triolein VLE at 313 K (vapor phase compositions). Experimental data [53,54] and CPA correlations using the binary parameter of Table 5 of the main article.

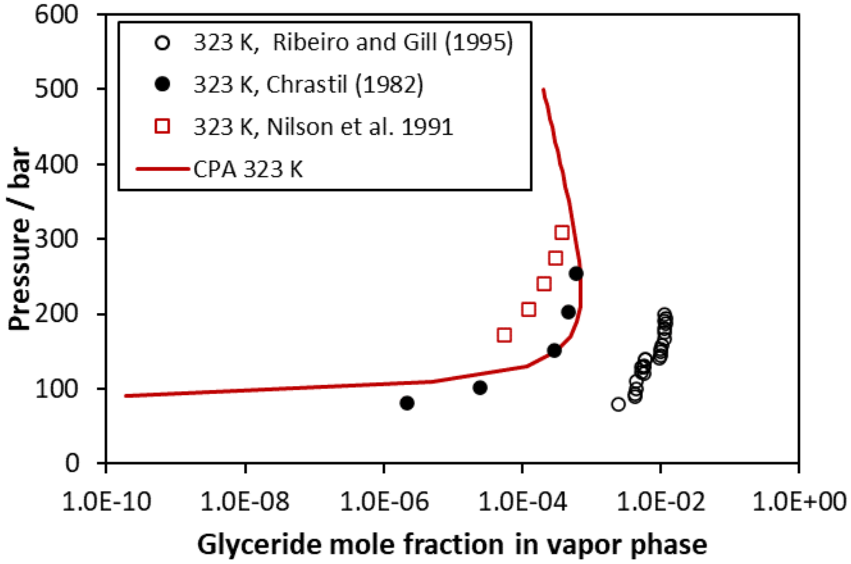

Figure S18. CO_2 – triolein VLE at 323 K (vapor phase compositions). Experimental data [50,53,54] and CPA correlations using the binary parameter of Table 5 of the main article.

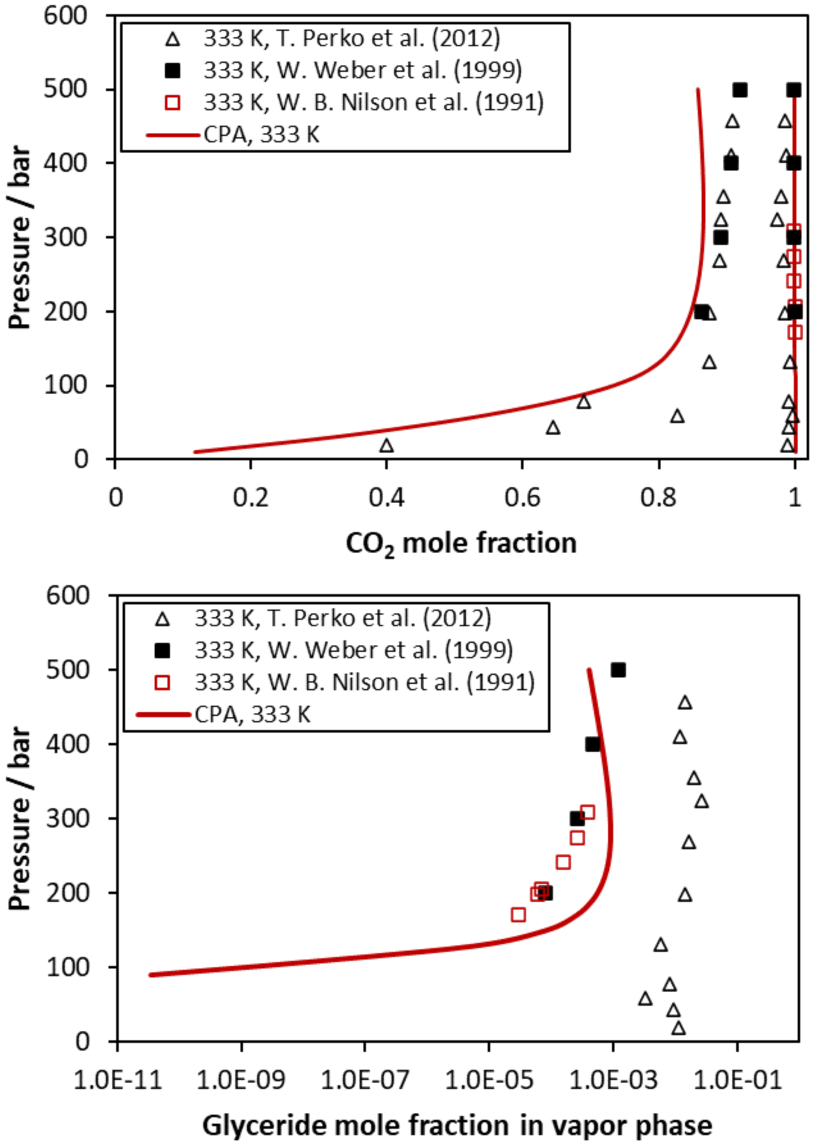

Figure S19. CO_2 – triolein VLE at 333 K. Experimental data [51-53] and CPA correlations using the binary parameter of Table 5 of the main article.

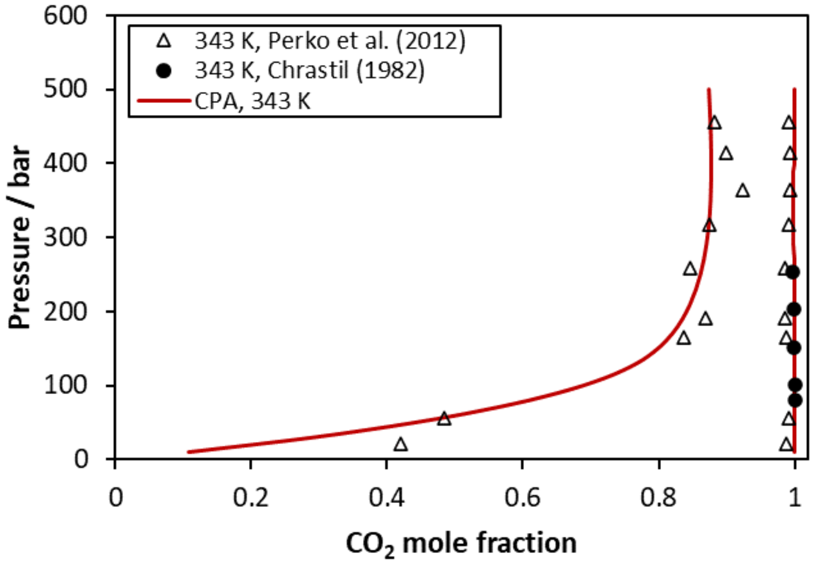

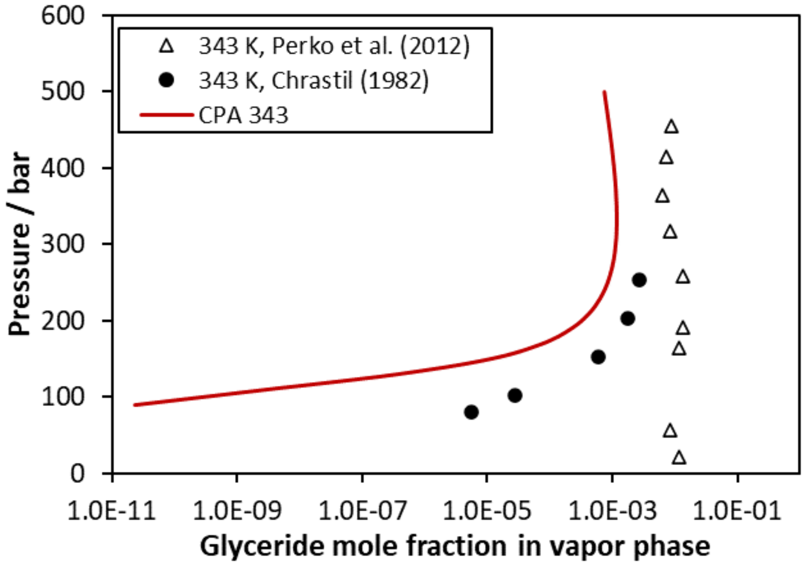

Figure S20. CO_2 – triolein VLE at 343 K. Experimental data [52,53] and CPA correlations using the binary parameter of Table 5 of the main article.
